# NNMT: Mean-Field Based Analysis Tools for Neuronal Network Models

**DOI:** 10.3389/fninf.2022.835657

**Published:** 2022-05-27

**Authors:** Moritz Layer, Johanna Senk, Simon Essink, Alexander van Meegen, Hannah Bos, Moritz Helias

**Affiliations:** ^1^Institute of Neuroscience and Medicine (INM-6) and Institute for Advanced Simulation (IAS-6) and JARA-Institute Brain Structure-Function Relationships (INM-10), Jülich Research Centre, Jülich, Germany; ^2^RWTH Aachen University, Aachen, Germany; ^3^Institute of Zoology, Faculty of Mathematics and Natural Sciences, University of Cologne, Cologne, Germany; ^4^Department of Physics, Faculty 1, RWTH Aachen University, Aachen, Germany

**Keywords:** mean-field theory, (spiking) neuronal network, integrate-and-fire neuron, open-source software, parameter space exploration, (hybrid) modeling, python, computational neuroscience

## Abstract

Mean-field theory of neuronal networks has led to numerous advances in our analytical and intuitive understanding of their dynamics during the past decades. In order to make mean-field based analysis tools more accessible, we implemented an extensible, easy-to-use open-source Python toolbox that collects a variety of mean-field methods for the leaky integrate-and-fire neuron model. The Neuronal Network Mean-field Toolbox (NNMT) in its current state allows for estimating properties of large neuronal networks, such as firing rates, power spectra, and dynamical stability in mean-field and linear response approximation, without running simulations. In this article, we describe how the toolbox is implemented, show how it is used to reproduce results of previous studies, and discuss different use-cases, such as parameter space explorations, or mapping different network models. Although the initial version of the toolbox focuses on methods for leaky integrate-and-fire neurons, its structure is designed to be open and extensible. It aims to provide a platform for collecting analytical methods for neuronal network model analysis, such that the neuroscientific community can take maximal advantage of them.

## 1. Introduction

Biological neuronal networks are composed of large numbers of recurrently connected neurons, with a single cortical neuron typically receiving synaptic inputs from thousands of other neurons (Braitenberg and Schüz, 1998; DeFelipe et al., 2002). Although the inputs of distinct neurons are integrated in a complex fashion, such large numbers of weak synaptic inputs imply that average properties of entire populations of neurons do not depend strongly on the contributions of individual neurons (Amit and Tsodyks, 1991). Based on this observation, it is possible to develop analytically tractable theories of population properties, in which the effects of individual neurons are averaged out and the complex, recurrent input to individual neurons is replaced by a self-consistent effective input (reviewed, e.g., in Gerstner et al., 2014). In classical physics terms (e.g., Goldenfeld, 1992), this effective input is called *mean-field*, because it is the self-consistent mean of a *field*, which here is just another name for the input the neuron is receiving. The term *self-consistent* refers to the fact that the population of neurons that receives the effective input is the same that contributes to this very input in a recurrent fashion: the population's output determines its input and vice-versa. The stationary statistics of the effective input therefore can be found in a self-consistent manner: the input to a neuron must be set exactly such that the caused output leads to the respective input.

Mean-field theories have been developed for many different kinds of synapse, neuron, and network models. They have been successfully applied to study average population firing rates (van Vreeswijk and Sompolinsky, 1996, 1998; Amit and Brunel, 1997b), and the various activity states a network of spiking neurons can exhibit, depending on the network parameters (Amit and Brunel, 1997a; Brunel, 2000; Ostojic, 2014), as well as the effects that different kinds of synapses have on firing rates (Fourcaud and Brunel, 2002; Lindner, 2004; Schuecker et al., 2015; Schwalger et al., 2015; Mattia et al., 2019). They have been used to investigate how neuronal networks respond to external inputs (Lindner and Schimansky-Geier, 2001; Lindner and Longtin, 2005), and they explain why neuronal networks can track external input on much faster time scales than a single neuron could (van Vreeswijk and Sompolinsky, 1996, 1998). Mean-field theories allow studying correlations of neuronal activity (Sejnowski, 1976; Ginzburg and Sompolinsky, 1994; Lindner et al., 2005; Trousdale et al., 2012) and were able to reveal why pairs of neurons in random networks, despite receiving a high proportion of common input, can show low output correlations (Hertz, 2010; Renart et al., 2010; Tetzlaff et al., 2012; Helias et al., 2014), which for example has important implication for information processing. They describe pair-wise correlations in network with spatial organization (Rosenbaum and Doiron, 2014; Rosenbaum et al., 2017; Dahmen et al., 2022) and can be generalized to correlations of higher orders (Buice and Chow, 2013). Mean-field theories were utilized to show that neuronal networks can exhibit chaotic dynamics (Sompolinsky et al., 1988; van Vreeswijk and Sompolinsky, 1996, 1998), in which two slightly different initial states can lead to totally different network responses, which has been linked to the network's memory capacity (Toyoizumi and Abbott, 2011; Schuecker et al., 2018). Most of the results mentioned above have been derived for networks of either rate, binary, or spiking neurons of a linear integrate-and-fire type. But various other models have been investigated with similar tools as well; for example, just to mention a few, Hawkes processes, non-linear integrate-and-fire neurons (Brunel and Latham, 2003; Fourcaud-Trocmé et al., 2003; Richardson, 2007, 2008; Grabska-Barwinska and Latham, 2014; Montbrió et al., 2015), or Kuramoto-type models (Stiller and Radons, 1998; van Meegen and Lindner, 2018). Additionally, there is an ongoing effort showing that many of the results derived for distinct models are indeed equivalent and that those models can be mapped to each other under certain circumstances (Ostojic and Brunel, 2011; Grytskyy et al., 2013; Senk et al., 2020).

Other theories for describing mean population rates in networks with spatially organized connectivity, based on taking a continuum limit, have been developed. These theories, known as neural field theories, have deepened our understanding of spatially and temporally structured activity patterns emerging in cortical networks, starting with the seminal work by Wilson and Cowan (1972, 1973), who investigated global activity patterns, and Amari (1975, 1977), who studied stable localized neuronal activity. They were successfully applied to explain hallucination patterns (Ermentrout and Cowan, 1979; Bressloff et al., 2001), as well as EEG and MEG rhythms (Nunez, 1974; Jirsa and Haken, 1996, 1997). The neural field approach has been used to model working memory (Laing et al., 2002; Laing and Troy, 2003), motion perception (Giese, 2012), cognition (Schöner, 2008), and more; for extensive reviews of the literature, we refer the reader to Coombes (2005), Bressloff (2012), and Coombes et al. (2014).

Clearly, analytical theories have contributed to our understanding of neuronal networks and they provide a plethora of powerful and efficient methods for network model analysis. Comparing the predictions of analytical theories to simulations, experimental data, or other theories necessitates a numerical implementation applicable to various network models, depending on the research question. Such an implementation is often far from straightforward and at times requires investing substantial time and effort. Commonly, such tools are implemented as the need arises, and their reuse is not organized systematically and restricted to within a single lab. This way, not only are effort and costs spent by the neuroscientific community duplicated over and over again, but also are many scientists deterred from taking maximal advantage of those methods although they might open new avenues for investigating their research questions.

In order to make analytical tools for neuronal network model analysis accessible to a wider part of the neuroscientific community, and to create a platform for collecting well-tested and validated implementations of such tools, we have developed the Python toolbox NNMT (Layer et al., 2021), short for Neuronal Network Mean-field Toolbox. We would like to emphasize that NNMT is not a simulation tool; NNMT is a collection of numerically solved mean-field equations that directly relate the parameters of a microscopic network model to the statistics of its dynamics. NNMT has been designed to fit the diversity of mean-field theories, and the key features we are aiming for are modularity, extensibility, and a simple usability. Furthermore, it features an extensive test suite to ensure the validity of the implementations as well as a comprehensive user documentation. The current version of NNMT mainly comprises tools for investigating networks of leaky integrate-and-fire neurons as well as some methods for studying binary neurons and neural field models. The toolbox is open-source and publicly available on GitHub.^1^

In the following, we present the design considerations that led to the structure and implementation of NNMT as well as a representative set of use cases. Section 2 first introduces its architecture. Section 3 then explains its usage by reproducing previously published network model analyses from Schuecker et al. (2015), Bos et al. (2016), Sanzeni et al. (2020), and Senk et al. (2020). Section 4 compares NNMT to other available toolboxes for neuronal network model analysis, discusses its use cases from a more general perspective, indicates current limitations and prospective advancements of NNMT, and explains how new tools can be contributed.

## 2. Workflows and Architecture

What are the requirements a package for collecting analytical methods for neuronal network model analysis needs to fulfill? To begin with, it should be adaptable and modular enough to accommodate many and diverse analytical methods while avoiding code repetition and a complex interdependency of package components. It should enable the application of the collected algorithms to various network models in a simple and transparent manner. It should make the tools easy to use for new users, while also providing experts with direct access to all parameters and options. Finally, the methods need to be thoroughly tested and well documented.

**Figure 1:**
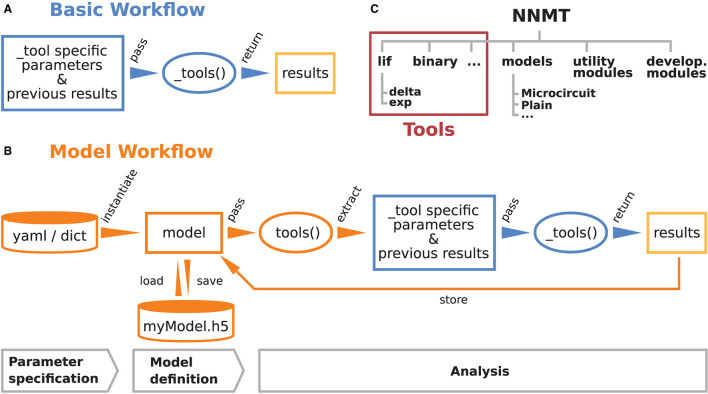
Structure and workflows of the Neuronal Network Mean-field Toolbox (NNMT). **(A)** Basic workflow: individual mean-field based analysis methods are implemented as functions, called _tools(), that can be used directly by explicitly passing the required arguments. **(B)** Model workflow: to facilitate the handling of parameters and results, they can be stored in a model class instance, which can be passed to a tool(), which wraps the basic workflow of the respective _tool(). **(C)** Structure of the Python package. In addition to the tool collection (red frame), containing the tools() and the _tools(), and pre-defined model classes, the package provides utility functions for handling parameter files and unit conversions, as well as software aiding the implementation of new methods.

These are the main considerations that guided the development of NNMT. Figures 1A,B illustrate how the toolbox can be used in to two different workflows, depending on the preferences and goals of the user. In the *basic workflow* the individual method implementations called *tools* are directly accessed, whereas the *model workflow* provides additional functionality for the handling of parameters and results.

### 2.1. Basic Workflow

The core of NNMT is a collection of low-level functions that take specific parameters (or pre-computed results) as input arguments and return analytical results of network properties. In Figure 1A, we refer to such basic functions as _tools(), as their names always start with an underscore. We term this lightweight approach of directly using these functions the basic workflow. The top part of Listing 1 demonstrates this usage; for example, the quantity to be computed could be the mean firing rate of a neuronal population and the arguments could be parameters which define neuron model and external drive. While the basic workflow gives full flexibility and direct access to every parameter of the calculation, it remains the user's responsibility to insert the arguments correctly, e.g., in the right units.

**Listing 1:**
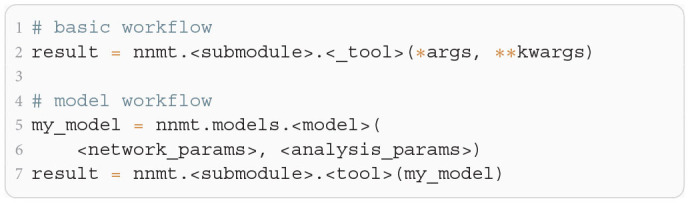
The two modes of using NNMT: In the basic workflow (top), quantities are calculated by passing all required arguments directly to the underscored tool functions available in the submodules of NNMT. In the model workflow (bottom), a model class is instantiated with parameter sets and the model instance is passed to the non-underscored tool functions which automatically extract the relevant parameters.

### 2.2. Model Workflow

The model workflow is a convenient wrapper of the basic workflow (Figure 1B). A *model* in this context is an object that stores a larger set of parameters and can be passed directly to a tool(), the non-underscored wrapper of the respective _tool(). The tool() automatically extracts the relevant parameters from the model, passes them as arguments to the corresponding core function _tool(), returns the results, and stores them in the model. The bottom part of Listing 1 shows how a model is initialized with parameters and then passed to a tool() function.

Models are implemented as Python classes and can be found in the submodule nnmt.models. We provide the class nnmt.models.Network as a parent class and a few child classes which inherit the generic methods and properties but are tailored to specific network models; custom models can be created straightforwardly. The parameters distinguish network parameters, which define neuron models and network connectivity, and analysis parameters; an example for an analysis parameter is a frequency range over which a function is evaluated. Upon model instantiation, parameter sets defining values and corresponding units are passed as Python dictionaries or yaml files. The model constructor takes care of reading in these parameters, computing dependent parameters from the imported parameters, and converting all units to SI units for internal computations. Consequently, the parameters passed as arguments and the functions for computing dependent parameters of a specific child class need to be aligned. This design encourages a clear separation between a concise set of base parameters and functionality that transforms these parameters to the generic (vectorized) format that the tools work with. To illustrate this, consider the weight matrix of a network of excitatory and inhibitory neuron populations in which all excitatory connections have the same weight and all inhibitory ones another weight. As argument one could pass just a tuple of two different weight values and the corresponding model class would take care of constructing the full weight matrix. This happens in the example presented in Section 3.2.2: The parameter file network_params_microcircuit.yaml contains the excitatory synaptic weight and the ratio of inhibitory to excitatory weights. On instantiation, the full weight matrix is constructed from these two parameters, following the rules defined in nnmt.models.Microcircuit.

When a tool() is called, it checks whether the provided model object contains all required parameters and previously computed results. Then the tool() extracts the required arguments, calls the respective _tool(), and caches and returns the result. If the user attempts to compute the same property twice, using identical parameters, the tool() will retrieve the already computed result from the model's cache and return that value. Results can be exported to an HDF5 file and also loaded.

Using the model workflow instead of the basic workflow comes with the initial overhead of choosing a suitable combination of parameters and a model class, but has the advantages of a higher level of automation with built-in mechanisms for checking correctness of input (e.g., regarding units), reduced redundancy, and the options to store and load results. Both modes of using the toolbox can also be combined.

### 2.3. Structure of the Toolbox

The structure of the Python package NNMT is depicted in Figure 1C. It is subdivided into submodules containing the tools (e.g., nnmt.lif.exp, or nnmt.binary), the model classes (nnmt.models), helper routines for handling parameter files and unit conversions, as well as modules that collect reusable code employed in implementations for multiple neuron models (cf. Section 4.4). The tools are organized in a modular, extensible fashion with a streamlined hierarchy. To give an example, a large part of the currently implemented tools apply to networks of leaky integrate-and-fire (LIF) neurons, and they are located in the submodule nnmt.lif. The mean-field theory for networks of LIF neurons distinguishes between neurons with instantaneous synapses, also called delta synapses, and those with exponentially decaying post-synaptic currents. Similarly, the submodule for LIF neurons is split further into the two submodules nnmt.lif.delta and nnmt.lif.exp. NNMT also collects different implementations for computing the same quantity using different approximations or numerics, allowing for a comparison of different approaches.

Apart from the core package, NNMT comes with an extensive online documentation,^2^ including a quickstart tutorial, all examples presented in this paper, a complete documentation of all tools, as well as a guide for contributors.

Furthermore, we provide an extensive test suite that validates the tools by checking them against previously published results and alternative implementations where possible. This ensures that future improvements of the numerics do not break the tools.

## 3. How to Use the Toolbox

In this section, we demonstrate the practical use of NNMT by replicating a variety of previously published results. The examples presented have been chosen to cover a broad range of common use cases and network models. We include analyses of both stationary and dynamic network features, as mean-field theory is typically divided into two parts: stationary theory, which describes time-independent network properties of systems in a stationary state, and dynamical theory, which describes time-dependent network properties. Additionally, we show how to use the toolbox to map a spiking to a simpler rate model, as well as how to perform a linear stability analysis. All examples, including the used parameter files, are part of the online documentation.^2^

### 3.1. Installation and Setup

The toolbox can be either installed using pip:


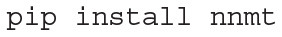


or by installing it directly from the repository, which is described in detail in the online documentation. After the installation, the module can be imported:


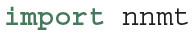


### 3.2. Stationary Quantities

#### 3.2.1. Response Nonlinearities

**Figure 2:**
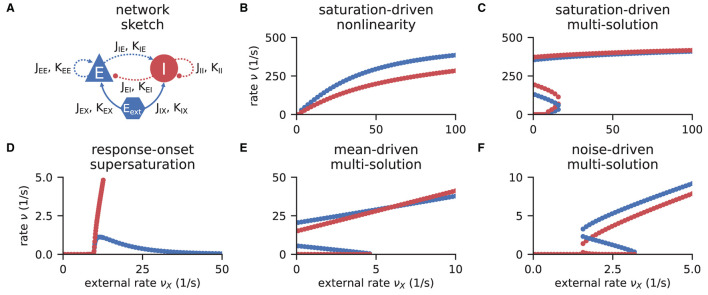
Response nonlinearities in EI-networks. **(A)** Network diagram with nodes and edges according to the graphical notation proposed by Senk et al. (in press). **(B–F)** Firing rate of excitatory (blue) and inhibitory (red) population for varying external input rate ν_*X*_. Specific choices for synaptic weights (***J***, ***J***_ext_) and in-degrees (***K***, ***K***_ext_) lead to five types of nonlinearities: **(B)** saturation-driven nonlinearity, **(C)** saturation-driven multi-solution, **(D)** response-onset supersaturation, **(E)** mean-driven multi-solution, and **(F)** noise-driven multi-solution. See Figure 8 in Sanzeni et al. (2020) for parameters.

Networks of excitatory and inhibitory neurons (EI networks, Figure 2A) are widely used in computational neuroscience (Gerstner et al., 2014), e.g., to show analytically that a balanced state featuring asynchronous, irregular activity emerges dynamically in a broad region of the parameter space (van Vreeswijk and Sompolinsky, 1996, 1998; Brunel, 2000; Hertz, 2010; Renart et al., 2010). Remarkably, such balance states emerge in inhibition dominated networks for a variety of neuron models if the indegree is large, *K* ≫ 1, and the weights scale as $J\propto 1/\sqrt{K}$ (Sanzeni et al., 2020; Ahmadian and Miller, 2021). Furthermore, in a balanced state, a network responds linearly to external input in the limit *K* → ∞ (van Vreeswijk and Sompolinsky, 1996, 1998; Brunel, 2000; Sanzeni et al., 2020; Ahmadian and Miller, 2021). How do EI networks of LIF neurons respond to external input at finite indegrees? Sanzeni et al. (2020) uncover five different types of nonlinearities in the network response depending on the network parameters. Here, we show how to use the toolbox to reproduce their result (Figures 2B–F).

The network consists of two populations, E and I, of identical LIF neurons with instantaneous (*delta*) synapses (Gerstner et al., 2014). The subthreshold dynamics of the membrane potential *V*_*i*_ of neuron *i* obeys


(1) $$\begin{array}{l} \tau_{\text{m}}{\dot{V}}_{i}=-V_{i}+RI_{i}, \end{array}$$

where τ_m_ denotes the membrane time constant, *R* the membrane resistance, and *I*_*i*_ the input current. If the membrane potential exceeds a threshold *V*_th_, a spike is emitted and the membrane voltage is reset to the reset potential *V*_0_ and clamped to this value during the refractory time τ_r_. After the refractory period, the dynamics continue according to Equation (1). For instantaneous synapses, the input current is given by


(2) $$\begin{array}{l} RI_{i}\left(t\right)=\tau_{\text{m}}\underset{j}{\sum }J_{ij}\underset{k}{\sum }\delta \left(t-t_{j,k}-d_{ij}\right), \end{array}$$

where *J*_*ij*_ is the synaptic weight from presynaptic neuron *j* to postsynaptic neuron *i* (with *J*_*ij*_ = 0 if there is no synapse), the *t*_*j,k*_ are the spike times of neuron *j*, and *d*_*ij*_ is a synaptic delay (in this example *d*_*ij*_ = *d* for all pairs of neurons). In total, there are *N*_E_ and *N*_I_ neurons in the respective populations. Each neuron is connected to a fixed number of randomly chosen presynaptic neurons (fixed in-degree); additionally, all neurons receive external input from independent Poisson processes with rate ν_X_. The synaptic weights and in-degrees of recurrent and external connections are population-specific:


(3) $$\begin{array}{l} J=\left(\begin{array}{cc} J_{\text{EE}} & -J_{\text{EI}}\\ J_{\text{IE}} & -J_{\text{II}} \end{array}\right),\;J_{\text{ext}}=\left(\begin{array}{c} J_{\text{EX}}\\ J_{\text{IX}} \end{array}\right),\\ K=\left(\begin{array}{cc} K_{\text{EE}} & K_{\text{EI}}\\ K_{\text{IE}} & K_{\text{II}} \end{array}\right),\;K_{\text{ext}}=\left(\begin{array}{c} K_{\text{EX}}\\ K_{\text{IX}} \end{array}\right). \end{array}$$

All weights are positive, implying an excitatory external input.

The core idea of mean-field theory is to approximate the input to a neuron as Gaussian white noise ξ(*t*) with mean 〈ξ(*t*)〉 = μ and noise intensity $\langle \xi \left(t\right)\xi \left(t^{\prime }\right)\rangle =\tau_{\text{m}}\sigma^{2}\delta \left(t-t^{\prime }\right)$. This approximation is well-suited for asynchronous, irregular network states (van Vreeswijk and Sompolinsky, 1996, 1998; Amit and Brunel, 1997b). For a LIF neuron driven by such Gaussian white noise, the firing rate is given by (Siegert, 1951; Tuckwell, 1988; Amit and Brunel, 1997b)


(4) $$\begin{array}{l} \phi \left(\mu ,\sigma \right)={\left(\tau_{\text{r}}+\tau_{\text{m}}\sqrt{\pi }\int_{{\tilde{V}}_{0}\left(\mu ,\sigma \right)}^{{\tilde{V}}_{\text{th}}\left(\mu ,\sigma \right)}e^{s^{2}}\left(1+\text{erf}\left(s\right)\right)\text{d}s\right)}^{-1}, \end{array}$$

where the rescaled reset- and threshold-voltages are


(5) $$\begin{array}{l} {\tilde{V}}_{0}\left(\mu ,\sigma \right)=\frac{V_{0}-\mu }{\sigma },\;{\tilde{V}}_{\text{th}}\left(\mu ,\sigma \right)=\frac{V_{\text{th}}-\mu }{\sigma }. \end{array}$$

The first term in Equation (4) is the refractory period and the second term is the mean first-passage time of the membrane voltage from reset to threshold. The mean and the noise intensity of the input to a neuron in a population *a* ∈ {*E, I*}, which control the mean first-passage time through Equation (5), are determined by (Amit and Brunel, 1997b)


(6) $$\begin{array}{l} \mu_{a}=\tau_{\text{m}}\left(J_{a\text{E}}K_{a\text{E}}\nu_{\text{E}}-J_{a\text{I}}K_{a\text{I}}\nu_{\text{I}}+J_{a\text{X}}K_{a\text{X}}\nu_{\text{X}}\right), \end{array}$$

(7) $$\begin{array}{l} \sigma_{a}^{2}=\tau_{\text{m}}\left(J_{a\text{E}}^{2}K_{a\text{E}}\nu_{\text{E}}+J_{a\text{I}}^{2}K_{a\text{I}}\nu_{\text{I}}+J_{a\text{X}}^{2}K_{a\text{X}}\nu_{\text{X}}\right), \end{array}$$

respectively, where each term reflects the contribution of one population, with the corresponding firing rates of the excitatory ν_E_, inhibitory ν_I_, and external population ν_X_. Note that we use the letters *i, j, k*, …  to index single neurons and *a, b, c*, …  to index neuronal populations. Both μ_*a*_ and σ_*a*_ depend on the firing rate of the neurons ν_*a*_, which is in turn given by Equation (4). Thus, one arrives at the self-consistency problem


(8) $$\begin{array}{l} \nu_{a}=\phi \left(\mu_{a},\sigma_{a}\right), \end{array}$$

which is coupled across the populations due to Equation (6) and Equation (7).

Our toolbox provides two algorithms to solve Equation (8): (1) Integrating the auxiliary ordinary differential equation (ODE) ${\dot{\nu }}_{a}=-\nu_{a}+\phi \left(\mu_{a},\sigma_{a}\right)$ with initial values ν_*a*_(0) = ν_*a*,0_ using scipy.integrate.solve_ivp (Virtanen et al., 2020) until it reaches a fixed point ${\dot{\nu }}_{a}=0$, where Equation (8) holds by construction. (2) Minimizing the quadratic deviation $\sum_{a}{\left[\nu_{a}-\phi \left(\mu_{a},\sigma_{a}\right)\right]}^{2}$, using the least squares (LSTSQ) solver scipy.optimize.least_squares (Virtanen et al., 2020) starting from an initial guess ν_*a*,0_. The ODE method is robust to changes in the initial values and hence a good first choice. However, it cannot find self-consistent solutions that correspond to an unstable fixed point of the auxiliary ODE (note that the stability of the auxiliary ODE does not indicate the stability of the solution). To this end, the LSTSQ method can be used. Its drawback is that it needs a good initial guess, because otherwise the found minimum might be a local one where the quadratic deviation does not vanish, $\sum_{a}{\left[\nu_{a}-\phi \left(\mu_{a},\sigma_{a}\right)\right]}^{2}>0$, and which accordingly does not correspond to a self-consistent solution, ν_*a*_ ≠ ϕ(μ_*a*_, σ_*a*_). A prerequisite for both methods is a numerical solution of the integral in Equation (4); this is discussed in Section A.1 in the Appendix.

The solutions of the self-consistency problem Equation (8) for varying ν_X_ and fixed ***J***, ***J***_ext_, ***K***, and ***K***_ext_ reveal the five types of response nonlinearities (Figure 2). Different response nonlinearities arise through specific choices of synaptic weights, ***J*** and ***J***_ext_, and in-degrees, ***K*** and ***K***_ext_, which suggests that already a simple EI-network possesses a rich capacity for nonlinear computations. Whenever possible, we use the ODE method and resort to the LSTSQ method only if the self-consistent solution corresponds to an unstable fixed point of the auxiliary ODE. Combining both methods, we can reproduce the first columns of Figure 8 in Sanzeni et al. (2020), where all five types of nonlinearities are presented.

In all cases, we chose appropriate initial values ν_*a*,0_ for either method. Note that an exploratory analysis is necessary if the stability properties of a network model are unknown, and potentially multiple fixed points are to be uncovered because there are, to the best of our knowledge, no systematic methods in *d* > 1 dimensions that provide all solutions of a nonlinear system of equations.

In Listing 2, we show a minimal example to produce the data shown in Figure 2B. After importing the function that solves the self-consistency Equation (8), we collect the neuron and network parameters in a dictionary. Then, we loop through different values for the external rate ν_*X*_ and determine the network rates using the ODE method, which is sufficient in this example. In Listing 2 and to produce Figure 2B, we use the basic workflow because only one isolated tool of NNMT (nnmt.lif.delta._firing_rates()) is employed, which requires only a few parameters defining the simple EI-network.

**Listing 2:**
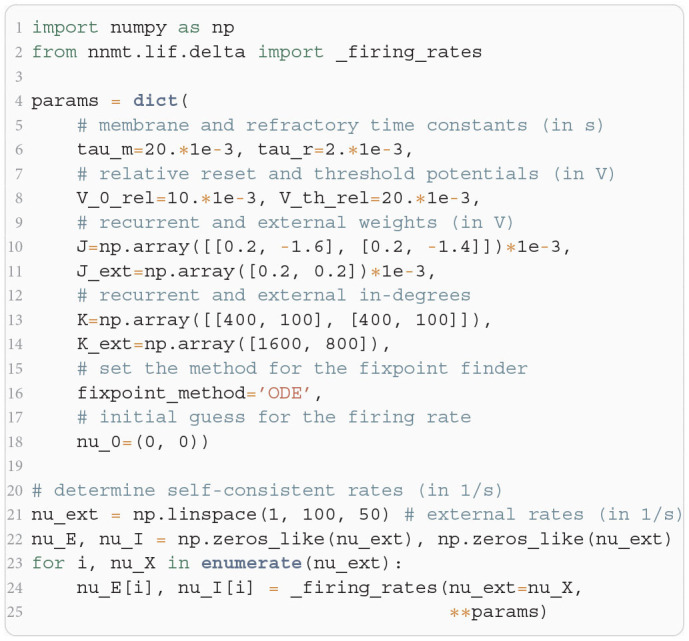
Example script to produce the data shown in Figure 2B using the ODE method (initial value ν_*a*,0_ = 0 for population *a* ∈ {*E, I*}).

#### 3.2.2. Firing Rates of Microcircuit Model

Here we show how to use the model workflow to calculate the firing rates of the cortical microcircuit model by Potjans and Diesmann (2014). The circuit is a simplified point neuron network model with biologically plausible parameters, which has been recently used in a number of other works: for example, to study network properties such as layer-dependent attentional processing (Wagatsuma et al., 2011), connectivity structure with respect to oscillations (Bos et al., 2016), and the effect of synaptic weight resolution on activity statistics (Dasbach, Tetzlaff, Diesmann, and Senk, 2021); to assess the performance of different simulator technologies such as neuromorphic hardware (van Albada et al., 2018) and GPUs (Knight and Nowotny, 2018; Golosio et al., 2021); to demonstrate forward-model prediction of local-field potentials from spiking activity (Hagen et al., 2016); and to serve as a building block for large-scale models (Schmidt et al., 2018).

**Figure 3:**
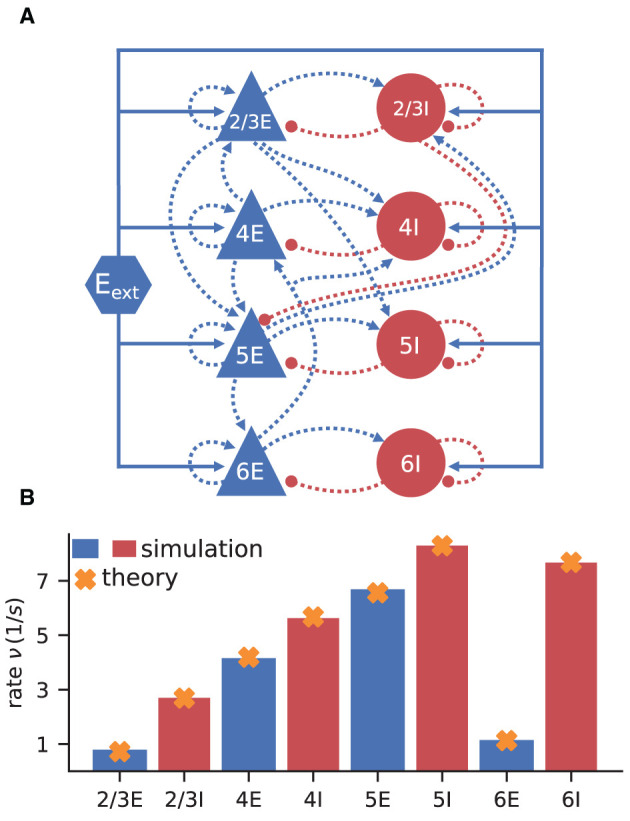
Cortical microcircuit model by Potjans and Diesmann (2014). **(A)** Network diagram (only the strongest connections are shown as in Figure 1 of the original publication). Same notation as in Figure 2A. **(B)** Simulation and mean-field estimate for average population firing rates using the parameters from Bos et al. (2016).

The model consists of eight populations of LIF neurons, corresponding to the excitatory and inhibitory populations of four cortical layers: 2/3E, 2/3I, 4E, 4I, 5E, 5I, 6E, and 6I (see Figure 3A). It defines the number of neurons in each population, the number of connections between the populations, the single neuron properties, and the external input. Simulations show that the model yields realistic firing rates for the different populations as observed in particular in the healthy resting-state of early sensory cortex (Potjans and Diesmann, 2014, Table 6).

In contrast to the EI-network model investigated in Section 3.2.1, the neurons in the microcircuit model have exponentially shaped post-synaptic currents: Equation (2) is replaced by Fourcaud and Brunel (2002)


(9) $$\begin{array}{l} \tau_{\text{s}}R\frac{\text{d}I_{i}}{\text{d}t}\left(t\right)=-RI_{i}\left(t\right)+\tau_{\text{m}}\underset{j}{\sum }J_{ij}\underset{k}{\sum }\delta \left(t-t_{j,k}-d_{ij}\right), \end{array}$$

with synaptic time constant τ_s_. Note that *J*_*ij*_ is a measure in volts here. As discussed in Section 3.2.1, in mean-field theory the second term, representing the neuronal input, is approximated by Gaussian white noise. The additional synaptic filtering leads to the membrane potential (Equation 1) receiving colored noise input. Fourcaud and Brunel (2002) developed a method for calculating the firing rate for this synapse type. They have shown that, if the synaptic time constant τ_s_ is much smaller than the membrane time constant τ_m_, the firing rate for LIF neurons with exponential synapses can be calculated using Equation (4) with shifted integration boundaries


(10) $$\begin{array}{l} {\tilde{V}}_{\text{cn},0}\left(\mu ,\sigma \right)={\tilde{V}}_{0}\left(\mu ,\sigma \right)+\frac{\alpha }{2}\sqrt{\frac{\tau_{\text{s}}}{\tau_{\text{m}}}},\\ {\tilde{V}}_{\text{cn,th}}\left(\mu ,\sigma \right)={\tilde{V}}_{\text{th}}\left(\mu ,\sigma \right)+\frac{\alpha }{2}\sqrt{\frac{\tau_{\text{s}}}{\tau_{\text{m}}}}, \end{array}$$

with the rescaled reset- and threshold-voltages from Equation (5) and $\alpha =\sqrt{2}|\zeta \left(1/2\right)|\approx 2.07$, where ζ(*x*) denotes the Riemann zeta function; the subscript cn stands for “colored noise”.

The microcircuit has been implemented as an NNMT model (nnmt.models.Microcircuit). We here use the parameters of the circuit as published in Bos et al. (2016) which is slightly differently parameterized than the original model (see Table A1 in the Appendix). The parameters of the model are specified in a yaml file, which uses Python-like indentation and a dictionary-style syntax. List elements are indicated by hyphens, and arrays can be defined as nested lists. Parameters with units can be defined by using the keys val and unit, whereas unitless variables can be defined without any keys. Listing 3 shows an example of how some of the microcircuit network parameters used here are defined. Which parameters need to be provided in the yaml file depends on the model used and is indicated in their respective docstrings.

**Listing 3:**
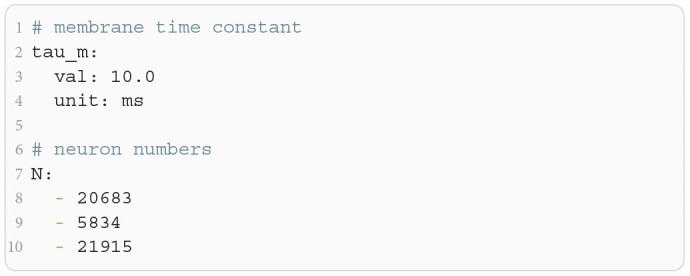
Some microcircuit network parameters defined in a yaml file. A dictionary-like structure with the keys val (value) and unit is used to define the membrane time constant, which is the same across all populations. The numbers of neurons in each population are defined as a list. Only the numbers for the first three populations are displayed.

Once the parameters are defined, a microcircuit model is instantiated by passing the respective parameter file to the model constructor; the units are automatically converted to SI units. Then the firing rates are computed. For comparison, we finally load the simulated rates from Bos et al. (2016):


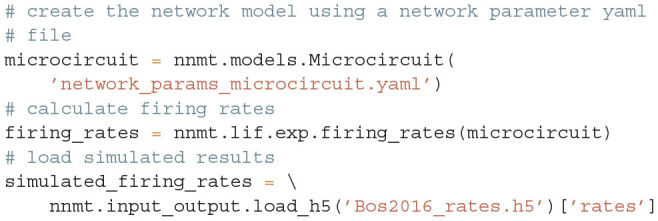


The simulated rates have been obtained by a numerical network simulation (for simulation details see Bos et al., 2016) in which the neuron populations are connected according to the model's original connectivity rule: “random, fixed total number with multapses (autapses prohibited)”, see Senk et al. (in press) as a reference for connectivity concepts. The term *multapses* refers to multiple connections between the same pair of neurons and *autapses* are self-connections; with this connectivity rule multapses can occur in a network realization but autapses are not allowed. For simplicity, the theoretical predictions assume a connectivity with a fixed in-degree for each neuron. Dasbach et al. (2021) show that simulated spike activity data of networks with these two different connectivity rules are characterized by differently shaped rate distributions (“reference” in their Figures 3d and 4d). In addition, the weights in the simulation are normally distributed while the theory replaces each distribution by its mean; this corresponds to the case *N*_bins_ = 1 in Dasbach et al. (2021). Nevertheless, our mean-field theoretical estimate of the average population firing rates is in good agreement with the simulated rates (Figure 3B).

### 3.3. Dynamical Quantities

#### 3.3.1. Transfer Function

One of the most important dynamical properties of a neuronal network is how it reacts to external input. A systematic way to study the network response is to apply an oscillatory external input current leading to a periodically modulated mean input μ(*t*) = μ+δμRe(e^iω*t*^) (cf. Equation 6), with fixed frequency ω, phase, and amplitude δμ, and observe the emerging frequency, phase, and amplitude of the output. If the amplitude of the external input is small compared to the stationary input, the network responds in a linear fashion: it only modifies phase and amplitude, while the output frequency equals the input frequency. This relationship is captured by the input-output transfer function *N*(ω) (Brunel and Hakim, 1999; Brunel et al., 2001; Lindner and Schimansky-Geier, 2001), which describes the frequency-dependent modulation of the output firing rate of a neuron population


$$\nu \left(t\right)=\nu +\text{Re}\left(N\left(\omega \right)\delta \mu {\text{e}}^{\text{i}\omega t}\right).$$

Note that in this section we only study the linear response to a modulation of the mean input, although in general, a modulation of the noise intensity (Equation 7) can also be included (Lindner and Schimansky-Geier, 2001; Schuecker et al., 2015). The transfer function *N*(ω) is a complex function: Its absolute value describes the relative modulation of the firing rate. Its phase, the angle relative to the real axis, describes the phase shift that occurs between input and output. We denote the transfer function for a network of LIF neurons with instantaneous synapses in linear-response approximation as


(11) $$\begin{array}{l} N\left(\omega \right)=\frac{\sqrt{2}\nu }{\sigma }\frac{1}{1+\text{i}\omega \tau_{\text{m}}}\frac{\Phi_{\omega }^{\prime }{|}_{\sqrt{2}{\tilde{V}}_{0}}^{\sqrt{2}{\tilde{V}}_{\text{th}}}}{\Phi_{\omega }{|}_{\sqrt{2}{\tilde{V}}_{0}}^{\sqrt{2}{\tilde{V}}_{\text{th}}}}, \end{array}$$

with the rescaled reset- and threshold-voltages ${\tilde{V}}_{0}$ and ${\tilde{V}}_{\text{th}}$ as defined in Equation (5) and $\Phi_{\omega }\left(x\right)=e^{\frac{x^{2}}{4}}U\left(\text{i}\omega \tau_{\text{m}}-\frac{1}{2},x\right)$ using the parabolic cylinder functions $U\left(\text{i}\omega \tau_{\text{m}}-\frac{1}{2},x\right)$ as defined in (Abramowitz and Stegun, 1974, Section 19.3) and (Olver et al., 2021, Section 12.2). $\Phi_{\omega }^{\prime }$ denotes the first derivative by *x*. A comparison of our notation and the transfer function given in Schuecker et al. (2015, Equation 29) can be found in Section A.2.1 in the Appendix.

For a neuronal network of LIF neurons with exponentially shaped post-synaptic currents, introduced in Section 3.2.2, Schuecker et al. (2014, 2015) show that an analytical approximation of the transfer function can be obtained by a shift of integration boundaries, akin to Equation (10):


(12) $$\begin{array}{l} N_{\text{cn}}\left(\omega \right)=\frac{\sqrt{2}\nu }{\sigma }\frac{1}{1+\text{i}\omega \tau_{\text{m}}}\frac{\Phi_{\omega }^{\prime }{|}_{\sqrt{2}{\tilde{V}}_{\text{cn,}0}}^{\sqrt{2}{\tilde{V}}_{\text{cn,th}}}}{\Phi_{\omega }{|}_{\sqrt{2}{\tilde{V}}_{\text{cn,}0}}^{\sqrt{2}{\tilde{V}}_{\text{cn,th}}}}. \end{array}$$

To take into account the effect of the synaptic dynamics, we include an additional low-pass filter:


(13) $$\begin{array}{l} N_{\text{cn,s}}\left(\omega \right)=N_{\text{cn}}\left(\omega \right)\frac{1}{1+\text{i}\omega \tau_{\text{s}}}. \end{array}$$

If the synaptic time constant is much smaller than the membrane time constant (τ_s_ ≪ τ_m_), an equivalent expression for the transfer function is obtained by a Taylor expansion around the original boundaries (cf. Schuecker et al. 2015, Equation 30). The toolbox implements both variants and offers choosing between them by setting the argument method of nnmt.lif.exp.transfer_function to either shift or taylor.

**Figure 4:**
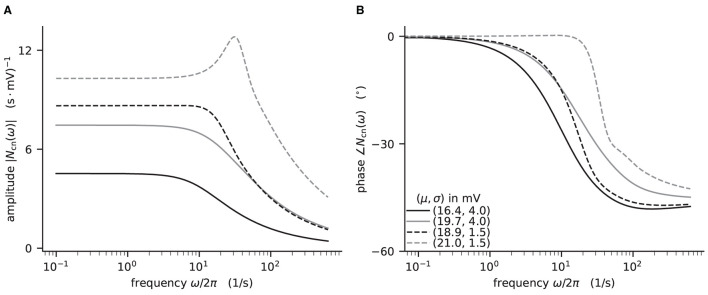
Colored-noise transfer function *N*_cn_ of LIF model in different regimes. **(A)** Absolute value and **(B)** phase of the “shift” version of the transfer function as a function of the log-scaled frequency. Neuron parameters are set to *V*_th_ = 20 mV, *V*_0_ = 15 mv, τ_m_ = 20 ms, and τ_s_ = 0.5 ms. For given noise intensities of input current, σ = 4 mV (solid line) and σ = 1.5 mV (dashed line), the mean input μ is chosen such that firing rates ν = 10 Hz (black) and ν = 30 Hz (gray) are obtained.

Here, we demonstrate how to calculate the analytical “shift version” of the transfer function for different means and noise intensities of the input current (see Figure 4) and thereby reproduce Figure 4 in Schuecker et al. (2015).

The crucial parts for producing Figure 4 using NNMT are shown in Listing 4 for one example combination of mean and noise intensity of the input current. Instead of using the model workflow with nnmt.lif.exp.transfer_function, we here employ the basic workflow, using nnmt.lif.exp._transfer_function directly. This allows changing the mean input and its noise intensity independently of a network model's structure, but requires two additional steps: First, the necessary parameters are loaded from a yaml file, converted to SI units and then stripped off the units using the utility function nnmt.utils._convert_to_si_and_strip_units. Second, the analysis frequencies are defined manually. In this example we choose logarithmically spaced frequencies, as we want to plot the results on a log-scale. Finally, the complex-valued transfer function is calculated and then split into its absolute value and phase. Figure 4 shows that the transfer function acts as a low-pass filter that suppresses the amplitude of high frequency activity, introduces a phase lag, and can lead to resonance phenomena for certain configurations of mean input current and noise intensity.

**Listing 4:**
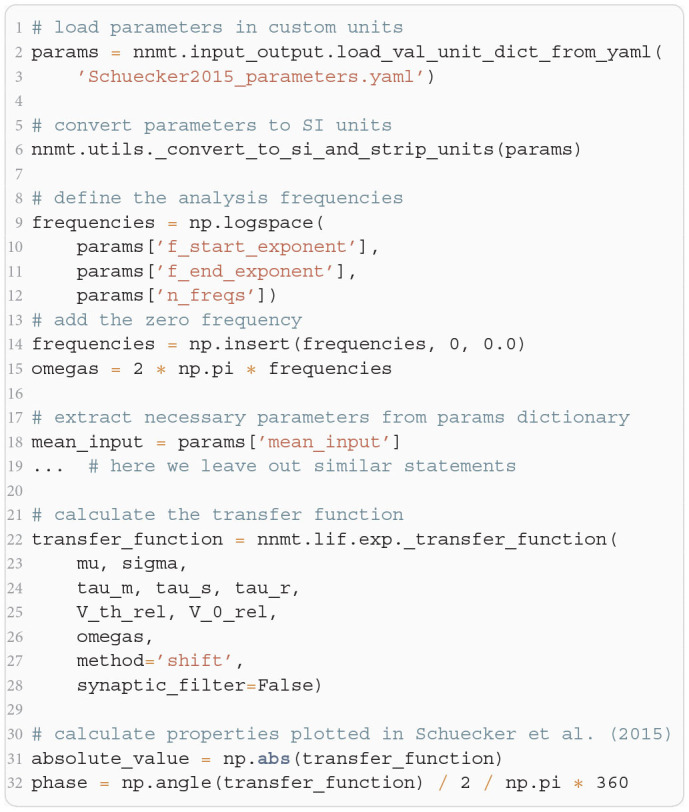
Example script for computing a transfer function shown in Figure 4 using the method of shifted integration boundaries.

The replication of the results from Schuecker et al. (2015) outlined here is also used in the integration tests of the toolbox. Note that the implemented analytical form of the transfer function by Schuecker et al. (2015) is an approximation for low frequencies, and deviations from a simulated ground truth are expected for higher frequencies (ω/2π ≳ 100 Hz at the given parameters).

#### 3.3.2. Power Spectrum

Another frequently studied dynamical property is the power spectrum, which describes how the power of a signal is distributed across its different frequency components, revealing oscillations of the population activity. The power is the Fourier transformed auto-correlation of the population activities (c.f. Bos et al. 2016, Equations 16-18). Linear response theory on top of a mean-field approximation, allows computing the power, dependent on the network architecture, the stationary firing rates, and the neurons' transfer function (Bos et al., 2016). The corresponding analytical expression for the power spectra of population *a* at angular frequency ω is given by the diagonal elements of the correlation matrix


(14) $$\begin{array}{l} P_{a}\left(\omega \right)=C_{aa}\left(\omega \right)\\ \;={\left[{\left(1-{\tilde{\text{M}}}_{\text{d}}\left(\omega \right)\right)}^{-1}\text{diag}\left(\nu ⊘n\right){\left(1-{\tilde{\text{M}}}_{\text{d}}\left(-\omega \right)\right)}^{-\text{T}}\right]}_{aa}, \end{array}$$

with ⊘ denoting the elementwise (Hadamard) division, the effective connectivity matrix ${\tilde{M}}_{\text{d}}\left(\omega \right)=\tau_{\text{m}}N_{\text{cn,s}}\left(\omega \right)\cdot J⊙K⊙D\left(\omega \right)$, where the dot denotes the scalar product, while ⊙ denotes the elementwise (Hadamard) product, the mean population firing rates ***ν***, and the numbers of neurons in each population ***n***. The effective connectivity combines the static, anatomical connectivity ***J*** ⊙ ***K***, represented by synaptic weight matrix ***J*** and in-degree matrix ***K***, and dynamical quantities, represented by the transfer functions *N*_cn,s,*a*_(ω) (Equation (13)), and the contribution of the delays in (Equation 13), represented by their Fourier transformed distributions *D*_*ab*_(ω) (cf. Bos et al. 2016, Equations 14, 15).

**Figure 5:**
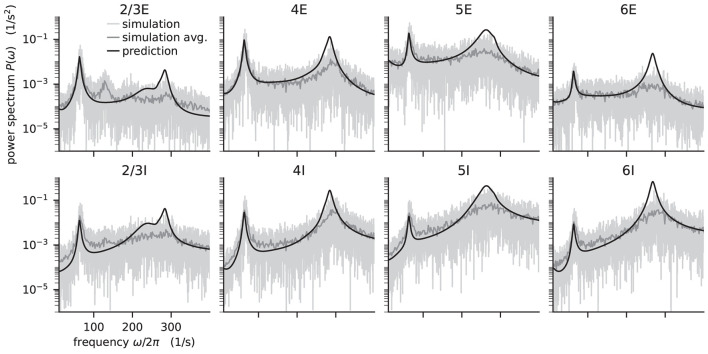
Power spectra of the population spiking activity in the adapted cortical microcircuit from Bos et al. (2016). The spiking activity of each population in a 10 s simulation of the model is binned with 1 ms resolution and the power spectrum of the resulting histogram is calculated by a fast Fourier transform (FFT; light gray curves). In addition, the simulation is split into 500 ms windows, the power spectrum calculated for each window and averaged across windows (gray curves). Black curves correspond to analytical prediction obtained with NNMT as described in Listing 5. The panels show the spectra for the excitatory (top) and inhibitory (bottom) populations within each layer of the microcircuit.

The modular structure in combination with the model workflow of this toolbox permits a step-by-step calculation of the power spectra, as shown in Listing 5. The inherent structure of the theory is emphasized in these steps: After instantiating the network model class with given network parameters, we determine the working point, which characterizes the statistics of the model's stationary dynamics. It is defined by the population firing rates, the mean, and the standard deviation of the input to a neuron of the respective population. This is necessary for determining the transfer functions. The calculation of the delay distribution matrix is then required for calculating the effective connectivity and to finally get an estimate of the power spectra. Figure 5 reproduces Figure 1E in Bos et al. (2016) and shows the spectra for each population of the adjusted version (see Table A1 in the Appendix) of the microcircuit model.

**Listing 5:**
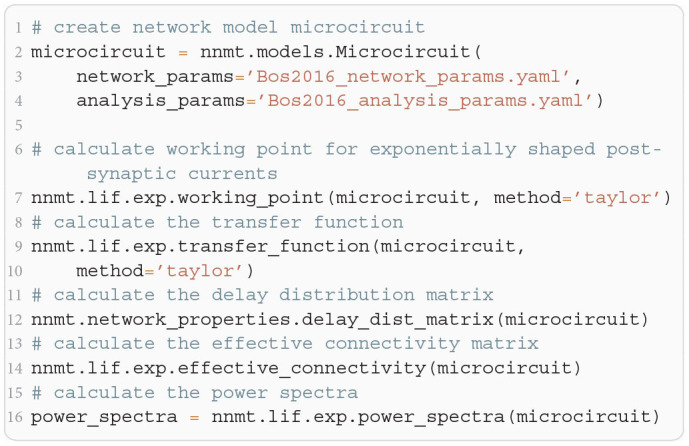
Example script to produce the theoretical prediction (black lines) shown in Figure 5B.

The numerical predictions obtained from the toolbox largely coincide with simulated data taken from the original publication (Bos et al., 2016) and reveal dominant oscillations of the population activities in the low-γ range around 63 Hz. Furthermore, faster oscillations with peak power around 300 Hz are predicted with higher magnitudes in the inhibitory populations 4I, 5I, and 6I.

The deviation between predicted and simulated power spectra seen at ~130 Hz in population 2/3E could be a harmonic of the correctly predicted, prominent 63 Hz peak; a non-linear effect not captured in linear response theory. Furthermore, the systematic overestimation of the power spectrum at large frequencies is explained by the limited validity of the analytical approximation of the transfer function for high frequencies.

#### 3.3.3. Sensitivity Measure

The power spectra shown in the previous section exhibit prominent peaks at certain frequencies, which indicate oscillatory activity. Naturally, this begs the question: which mechanism causes these oscillations? Bos et al. (2016) expose the crucial role that the microcircuit's connectivity plays in shaping the power spectra of this network model. They have developed a method called *sensitivity measure* to directly relate the influence of the anatomical connections, especially the in-degree matrix, on the power spectra.

The power spectrum of the *a*-th population *P*_*a*_(ω) receives a contribution from each eigenvalue λ_*b*_ of the effective connectivity matrix, $P_{a}\left(\omega \right)\propto 1/{\left(1-\lambda_{b}\left(\omega \right)\right)}^{2}$. Such a contribution consequently diverges as the complex-valued λ_*b*_ approaches 1+0i in the complex plane, which is referred to as the point of instability. This relation can be derived by replacing the effective connectivity matrix ${\tilde{\text{M}}}_{\text{d}}\left(\omega \right)$ in Equation (14) by its eigendecomposition. The sensitivity measure leverages this relationship and evaluates how a change in the in-degree matrix affects the eigenvalues of the effective connectivity and thus indirectly the power spectrum. Bos et al. (2016) introduce a small perturbation α_*cd*_ of the in-degree matrix, which allows writing the effective connectivity matrix as ${\hat{M}}_{ab}\left(\omega \right)=\left(1+\alpha_{cd}\delta_{ca}\delta_{db}\right){\tilde{M}}_{ab}\left(\omega \right)$, where we dropped the delay subscript d. The sensitivity measure *Z*_*b,cd*_(ω) describes how the *b*-th eigenvalue of the effective connectivity matrix varies when the *cd*-th element of the in-degree matrix is changed


(15) $$\begin{array}{l} Z_{b,cd}\left(\omega \right)={\frac{\partial \lambda_{b}\left(\omega \right)}{\partial \alpha_{cd}}|}_{\alpha_{cd=0}}=\frac{v_{b,c}{\tilde{M}}_{cd}u_{b,d}}{{\text{v}}_{b}^{\text{T}}\cdot {\text{u}}_{b}}, \end{array}$$

where $\frac{\partial \lambda_{b}\left(\omega \right)}{\partial \alpha_{cd}}$ is the partial derivative of the eigenvalue with respect to a change in connectivity, ${\text{v}}_{b}^{\text{T}}$ and **u**_*b*_ are the left and right eigenvectors of $\tilde{M}$ corresponding to eigenvalue λ_*b*_(ω).

The complex sensitivity measure can be understood in terms of two components: ${\text{Z}}_{b}^{\text{amp}}$ is the projection of the matrix **Z**_*b*_ onto the direction in the complex plane defined by 1 − λ_*b*_(ω); it describes how, when the in-degree matrix is perturbed, the complex-valued λ_*b*_(ω) moves toward or away from the instability 1 + 0i, and consequently how the amplitude of the power spectrum at frequency ω increases or decreases. ${\text{Z}}_{b}^{\text{freq}}$ is the projection onto the perpendicular direction and thus describes how the peak frequency of the power spectrum changes with the perturbation of the in-degree matrix. For a visualization of these projections, refer to Figure 5B in Bos et al. (2016).

The toolbox makes this intricate measure accessible by supplying two tools: After computing the required working point, transfer function, and delay distribution, the tool nnmt.lif.exp.sensitivity_measure computes the sensitivity measure at a given frequency for one specific eigenvalue. By default, this is the eigenvalue which is closest to the instability 1 + 0i. To perform the computation, we just need to add one line to Listing 5:


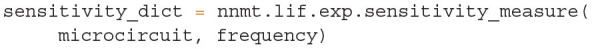


The result is returned in form of a dictionary that contains the sensitivity measure and its projections. The tool nnmt.lif.exp.sensitivity_measure_all_eigenmodes wraps that basic function and calculates the sensitivity measure for all eigenvalues at the frequency for which each eigenvalue is closest to instability.

**Figure 6:**
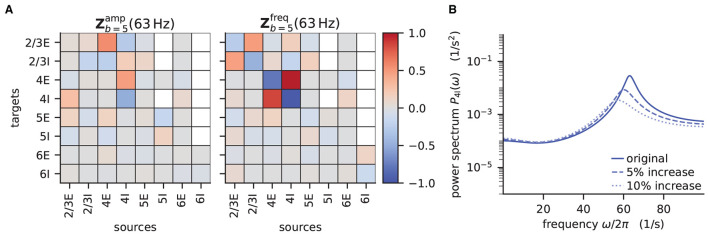
Sensitivity measure at low-γ frequency and corresponding power spectrum of microcircuit with adjusted connectivity. **(A)** Sensitivity measure of one eigenmode of the effective connectivity relevant for low-γ oscillations. The sensitivity measure for this mode is evaluated at the frequency where the corresponding eigenvalue is closest to the point of instability 1 + 0i in complex plane. $Z_{b}^{\text{amp}}\left(\omega \right)$ (left subpanel) visualizes the influence of a perturbation of a connection on the peak amplitude of the power spectrum. $Z_{b}^{\text{freq}}\left(\omega \right)$ (right subpanel) shows the impact on the peak frequency. Non-existent connections are masked white. **(B)** Mean-field prediction of power spectrum of population 4I with original connectivity parameters (solid line), 5% increase (dashed line) and 10% increase (dotted line) in connections *K*_4I → 4I_. The increase in inhibitory input to population 4I was counteracted by an increase of the excitatory external input *K*_ext → 4I_ to maintain the working point.

According to the original publication (Bos et al., 2016), the peak around 63Hz has contributions from one eigenvalue of the effective connectivity matrix. Figure 6 shows the projections of the sensitivity measure at the frequency for which this eigenvalue is closest to the instability, as illustrated in Figure 4 of Bos et al. (2016). The sensitivity measure returns one value for each connection between populations in the network model. For ${\text{Z}}_{b}^{\text{amp}}$ a negative value indicates that increasing the in-degrees of a specific connection causes the amplitude of the power spectrum at the evaluated frequency to drop. If the value is positive, the amplitude is predicted to grow as the in-degrees increase. Similarly, for positive ${\text{Z}}_{b}^{\text{freq}}$ the frequency of the peak in the power spectrum shifts toward higher values as in-degrees increase, and vice versa. The main finding in this analysis is that the low-γ peak seems to be affected by excitatory-inhibitory loops in layer 2/3 and layer 4.

To decrease the low–γ peak in the power spectrum, one could therefore increase the 4I to 4I connections (cp. Figure 6A):


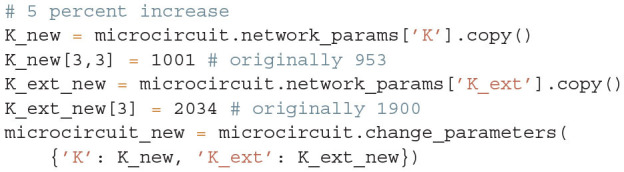


and calculate the power spectrum as in Listing 5 again to validate the change. Note that a change in connectivity leads to a shift in the working point. We are interested in the impact of the modified connectivity on the fluctuation dynamics at the same working point and thus need to counteract the change in connectivity by adjusting the external input. In the chosen example this is ensured by satisfying *J*_4I → 4*I*_Δ*K*_4I → 4*I*_ν_4I_ = −*J*_ext → 4I_Δ*K*_ext → 4I_ν_ext_, which yields $\Delta K_{\text{ext}\to \text{4I}}=-\frac{J_{\text{4I}\to 4I}\Delta K_{\text{4I}\to 4I}\nu_{\text{4I}}}{J_{\text{ext}\to \text{4I}}\nu_{\text{ext}}}$.

If several eigenvalues of the effective connectivity matrix influence the power spectra in the same frequency range, adjustments of the connectivity are more involved. This is because a change in connectivity would inevitably affect all eigenvalues simultaneously. Further care has to be taken because the sensitivity measure is subject to the same constraints as the current implementation of the transfer function, which is only valid for low frequencies and enters the sensitivity measure directly.

### 3.4. Fitting Spiking to Rate Model and Predicting Pattern Formation

**Figure 7:**
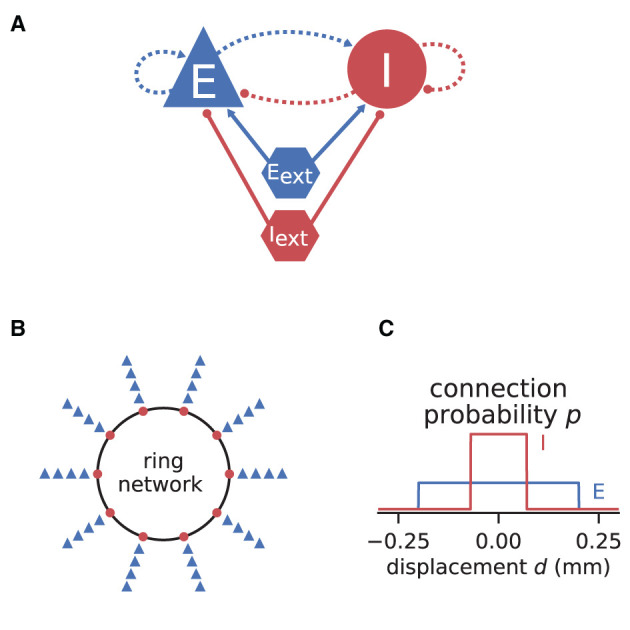
Illustrations of spiking network model by Senk et al. (2020). **(A)** Excitatory and inhibitory neuronal populations randomly connected with fixed in-degree and multapses allowed (autapses prohibited). External excitatory and inhibitory Poisson drive to all neurons. Same notation as in Figure 2A. **(B)** One inhibitory and four excitatory neurons per grid position on a one-dimensional domain with periodic boundary conditions (ring network). **(C)** Normalized, boxcar-shaped connection probability with wider excitation than inhibition; the grid spacing is here 10^−3^ mm. For model details and parameters, see Tables II–IV of Senk et al. (2020); the specific values given in the caption of their Figure 6 are used throughout here.

If the neurons of a network are spatially organized and connected according to a distance-dependent profile, the spiking activity may exhibit pattern formation in space and time, including wave-like phenomena. Senk et al. (2020) set out to scrutinize the non-trivial relationship between the parameters of such a network model and the emerging activity patterns. The model they use is a two-population network of excitatory E and inhibitory I spiking neurons, illustrated in Figure 7. All neurons are of type LIF with exponentially shaped post-synaptic currents. The neuron populations are recurrently connected to each other and themselves and they receive additional external excitatory E_ext_ and inhibitory I_ext_ Poisson spike input of adjustable rate as shown in Figure 7A. The spatial arrangement of neurons on a ring is illustrated in Figure 7B and the boxcar-shaped connectivity profiles in Figure 7C.

In the following, we consider a mean-field approximation of the spiking model with spatial averaging, that is a time and space continuous approximation of the discrete model as derived in Senk et al. (2020, Section E. Linearization of spiking network model). We demonstrate three methods used in the original study: First, Section 3.4.1 explains how a model can be brought to a defined state characterized by its working point. The working point is given by the mean μ and noise intensity σ of the input to a neuron, which are both quantities derived from network parameters and require the calculation of the firing rates. With the spiking model in that defined state, Section 3.4.2 then maps its transfer function to the one of a rate model. Section 3.4.3 finally shows that this working-point dependent rate model allows for an analytical linear stability analysis of the network accounting for its spatial structure. This analysis can reveal transitions to spatial and temporal oscillatory states which, when mapped back to the parameters of the spiking model, may manifest in distinct patterns of simulated spiking activity after a startup transient.

#### 3.4.1. Setting the Working Point by Changing Network Parameters

With network and analysis parameters predefined in yaml files, we set up a network model using the example model class Basic:


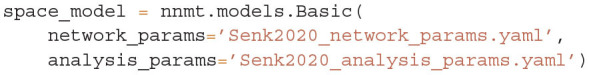


Upon initialization the given parameters are automatically converted into the format used by NNMT's tools. For instance, relative spike reset and threshold potentials are derived from the absolute values, connection strengths in units of volt are computed from the post-synaptic current amplitudes in ampere, and all values are scaled to SI units.

We aim to bring the network to a defined state by fixing the working point but also want to explore if the procedure of fitting the transfer function still works for different network states. For a parameter space exploration, we use a method to change parameters provided by the model class and scan through a number of different working points of the network. To obtain the required input for a target working point, we adjust the external excitatory and inhibitory firing rates accordingly; NNMT uses a vectorized version of the equations given in Senk et al. (2020, Appendix F: Fixing the working point) to calculate the external rates needed:


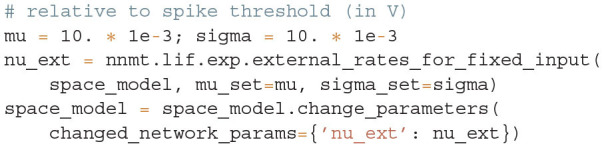


**Figure 8:**
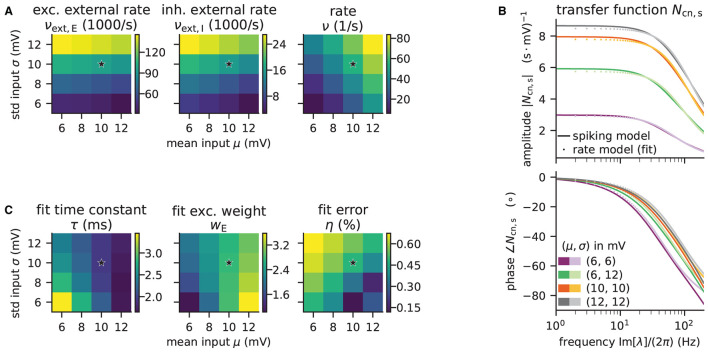
Network parameters and mean-field results from scanning through different working points. Working point (μ, σ) combines mean input μ and noise intensity of input σ. **(A)** External excitatory ν_ext,E_ and inhibitory ν_ext,I_ Poisson rates required to set (μ, σ) and resulting firing rates ν. **(B)** Transfer function *N*_cn,s_ of spiking model and fitted rate-model approximation with low-pass filter for selected (μ, σ) (top: amplitude, bottom: phase). **(C)** Fit results (time constants τ and excitatory weights *w*_E_) and fit errors η. The inhibitory weights are *w*_I_ = −*gw*_E_ with *g* = 5. Star marker in panels **(A)** and **(C)** denotes target working point (10, 10) mV. Similar displays as in Senk et al. (2020, Figure 5).

The implementation uses only one excitatory and one inhibitory Poisson source to represent the external input rates which typically originate from a large number of external source neurons. These two external sources are connected to the network with the same relative inhibition *g* as used for the internal connections. The resulting external rates for different choices of (μ, σ) are color-coded in the first two plots of Figure 8A. The third plot shows the corresponding firing rates of the neurons, which are stored in the results of the model instance when computing the working point explicitly:


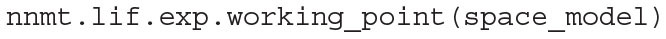


Although the external rates are substantially higher than the firing rates, since a neuron is recurrently connected to hundreds of neurons, the total external and recurrent inputs are of the same order.

#### 3.4.2. Parameter Mapping by Fitting the Transfer Function

We map the parameters of the spiking model to a corresponding rate model by, first, computing the transfer function ***N***_cn,s_ given in Equation (13) of the spiking model, and second, fitting the simpler transfer function of the rate model, for details see Senk et al. (2020, Section F. Comparison of neural-field and spiking models). The dynamics of the rate model can be written as a differential equation for the linearized activity *r*_*a*_ with populations *a, b* ∈ {E, I}:


(16) $$\begin{array}{l} \tau \frac{\text{d}}{\text{d}t}r_{a}\left(t\right)=-r_{a}\left(t\right)+\underset{b}{\sum }w_{b}r_{b}\left(t-d\right) \end{array}$$

with the delay *d*; τ is the time constant and *w*_*b*_ are the unitless weights that only depend on the presynaptic population. The transfer function is just the one of a low-pass filter, *N*_LP_ = 1/(1 + λτ), where λ is the frequency in Laplace domain. The tool to fit the transfer function requires that the actual transfer function ***N***_cn,s_ has been computed beforehand and fits *N*_LP_***w*** to τ_m_***N***_cn,s_·***J*** ⊙ ***K*** for the same frequencies together with τ, ***w***, and the combined fit error η:


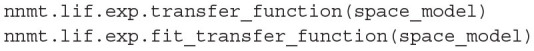


The absolute value of the transfer function is fitted with non-linear least-squares using the solver scipy.optimize.curve_fit. Figure 8B illustrates the amplitude and phase of the transfer function and its fit for a few (μ, σ) combinations. The plots of Figure 8C show the fitted time constants, the fitted excitatory weight, and the combined fit error. The inhibitory weight is proportional to the excitatory one in the same way as the post-synaptic current amplitudes.

#### 3.4.3. Linear Stability Analysis of Spatially Structured Model With Delay

Sections 3.4.1 and 3.4.2 considered a mean-field approximation of the spiking model without taking space into account. In the following, we assume a spatial averaging of the discrete network depicted in Figure 7 and introduce the spatial connectivity profiles *p*_*a*_(*x*). Changing Equation (16) to the integro-differential equation


(17) $$\begin{array}{l} \tau \frac{\partial }{\partial t}r_{a}\left(x,t\right)=-r_{a}\left(x,t\right)+\underset{b}{\sum }w_{b}\int_{-\infty }^{\infty }p_{b}\left(x-y\right)r_{b}\left(y,t-d\right)\text{d}y \end{array}$$

yields a neural field model defined in continuous space *x*. This model lends itself to analytical linear stability analysis, as described in detail in Senk et al. (2020, Section A. Linear stability analysis of a neural-field model). In brief, we analyze the stability of a fixed-point solution to this differential equation system which, together with parameter continuation methods and bifurcation analysis, allows us to determine points in parameter space where transitions from homogeneous steady states to oscillatory states can occur. These transitions are given as a function of a bifurcation parameter, here the constant delay *d*, which is the same for all connections. The complex-valued, temporal eigenvalue λ of the linearized time-delay system is an indicator for the system's overall stability and can serve as a predictor for temporal oscillations, whereas the spatial oscillations are characterized by the real-valued wave number *k*. Solutions that relate λ and *k* with the model parameters are given by a characteristic equation, which in our case reads (Senk et al., 2020, Equation 7):


(18) $$\begin{array}{l} \lambda_{B}\left(k\right)=-\frac{1}{\tau }+\frac{1}{d}W_{B}\left(c\left(k\right)\frac{d}{\tau }{\text{e}}^{\frac{d}{\tau }}\right), \end{array}$$

with the time constant of the rate model τ, the multi-valued Lambert *W*_*B*_ function^3^ on branch *B* (Corless et al., 1996), and the effective connectivity profile *c*(*k*), which combines the weights *w*_*b*_ and the Fourier transforms of the spatial connectivity profiles. Note that the approach generalizes from the boxcar-shaped profiles used here to any symmetric probability density function. NNMT provides an implementation to solve this characteristic equation in its linear_stability module using the spatial module for the profile:


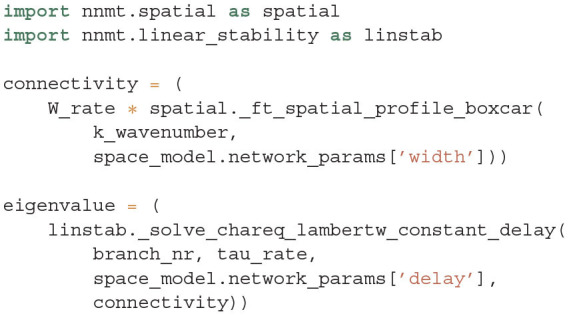


**Figure 9:**
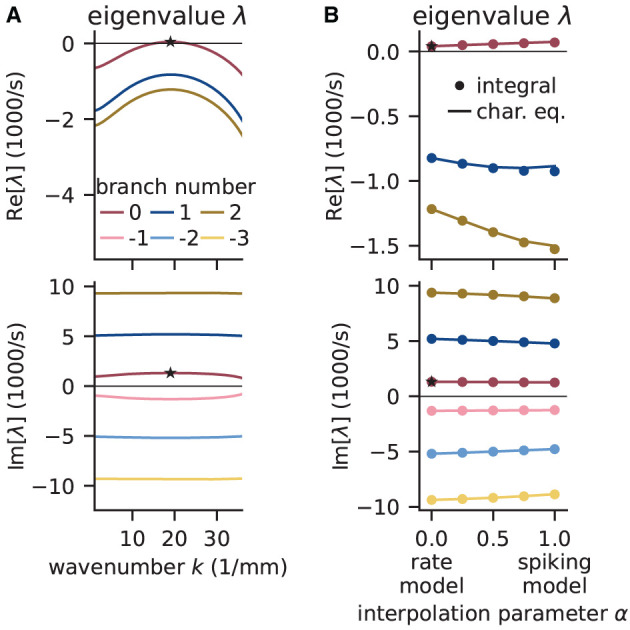
Linear stability analysis of spatially structured network model. **(A)** Analytically exact solution for real (top) and imaginary (bottom) part of eigenvalue λ vs. wavenumber *k* using rate model derived by fit of spiking model at working point (μ, σ) = (10, 10) mV. Color-coded branches of Lambert *W*_*B*_ function; maximum real eigenvalue (star marker) on principal branch (*B* = 0). **(B)** Linear interpolation between rate (α = 0) and spiking model (α = 1) by numerical integration of Senk et al. (2020, Equation 30) (solid line) and by numerically solving the characteristic equation in Senk et al. (2020, Equation 29) (circular markers). Star markers at same data points as in **(A)**. Similar displays as in Senk et al. (2020, Figure 6).

Figure 9A shows that the computed eigenvalues come for the given network parameters in complex conjugate pairs. The branch with the largest real part is the principal branch (*B* = 0). Temporal oscillations are expected to occur if the real part of the eigenvalue on the principal branch becomes positive; the oscillation frequency can then be read off the imaginary part of that eigenvalue. In this example, the largest eigenvalue λ^*^ on the principal branch has a real part that is just above zero. There exists a supercritical Hopf bifurcation and the delay as the bifurcation parameter is chosen large enough such that the model is just beyond the bifurcation point separating the stable from the instable state. The respective wave number k^*^ is positive, which indicates spatial oscillations as well. The oscillations in both time and space predicted for the rate model imply that the activity of the corresponding spiking model might exhibit wave trains, i.e., temporally and spatially periodic patterns. The predicted propagation speed of the wave trains is given by the phase velocity Im[λ^*^]/k^*^.

To determine whether the results obtained with the rate model are transferable to the spiking model, Figure 9B interpolates the analytical solutions of the rate model [α = 0, evaluating Equation (18)] to solutions of the spiking model (α = 1, accounting for the transfer function ***N***_cn,s_), which can only be computed numerically. Thus, the parameter α interpolates between the characteristic equations of these two models which primarily differ in their transfer function; for details see Senk et al. (2020, Section F.2 Linear interpolation between the transfer functions). Since the eigenvalues estimated this way show only little differences between rate and spiking model, we conclude that predictions from the rate model should hold also for the spiking model in the parameter regime tested. Following the argument of Senk et al. (2020), the predicted pattern formation could next be tested in a numerical simulation of the discrete spiking network model. Their Figure 7c for the delay *d* = 1.5 ms shows such results with the same parameters as used here; this figure also illustrates transitions from homogeneous states to oscillatory states by changing the delay (panels b, c, and e).

## 4. Discussion

Mean-field theory grants important insights into the dynamics of neuronal networks. However, the lack of a publicly available numerical implementation for most methods entails a significant initial investment of time and effort prior to any scientific investigations. In this paper, we present the open-source toolbox NNMT, which currently focuses on methods for LIF neurons but is intended as a platform for collecting standard implementations of various neuronal network model analyses based on mean-field theory that have been thoroughly tested and validated by the neuroscientific community (Riquelme and Gjorgjieva, 2021). As use cases, we reproduce known results from the literature: the non-linear relation between the firing rates and the external input of an E-I-network (Sanzeni et al., 2020), the firing rates of a cortical microcircuit model, its response to oscillatory input, its power spectrum, and the identification of the connections that predominantly contribute to the model's low frequency oscillations (Schuecker et al., 2015; Bos et al., 2016), and pattern formation in a spiking network, analyzed by mapping it to a rate model and conducting a linear stability analysis (Senk et al., 2020).

In the remainder of the discussion, we compare NNMT to other tools suited for network model analysis. We expand on the different use cases of NNMT and also point out the inherent limitations of analytical methods for neuronal network analysis. We conclude with suggestions on how new tools can be added to NNMT and how the toolbox may grow and develop in the future.

### 4.1. Comparison to Other Tools

There are various approaches and corresponding tools that can help to gain a better understanding of a neuronal network model. There are numerous simulators that numerically solve the dynamical equations for concrete realizations of a network model and all its stochastic components, often focusing either on the resolution of single-neurons, for example NEST (Gewaltig and Diesmann, 2007), Brian (Stimberg et al., 2019), or Neuron (Hines and Carnevale, 2001), or on the population level, for example TheVirtualBrain (Sanz Leon et al., 2013). Similarly, general-purpose dynamical system software like XPPAUT (Ermentrout, 2002) can be used. Simulation tools, like DynaSim (Sherfey et al., 2018), come with enhanced functionality for simplifying batch analysis and parameter explorations. All these tools yield time-series of activity, and some of them even provide the methods for analyzing the generated data. However, simulations only indirectly link a model's parameters with its activity: to gain an understanding of how a model's parameters influence the statistics of their activity, it is necessary to run many simulations with different parameters and analyze the generated data subsequently.

Other approaches provide a more direct insight into a model's behavior on an abstract level: TheVirtualBrain and the Brain Dynamics Toolbox (Heitmann et al., 2018), for example, allow plotting a model's phase space vector field while the parameters can be changed interactively, allowing for exploration of low-dimensional systems defined by differential equations without the need for simulations. XPPAUT has an interface to AUTO-07P (Doedel and Oldeman, 1998), a software for performing numerical bifurcation and continuation analysis. It is worth noting that such tools are limited to models that are defined in terms of differential equations. Models specified in terms of update rules, such as binary neurons, need to be analyzed differently, for example using mean-field theory.

A third approach is to simplify the model analytically and simulate the simplified version. The simulation platform DiPDE^4^ utilizes the population density approach to simulate the statistical evolution of a network model's dynamics. Schwalger et al. (2017) start from a microscopic model of generalized integrate-and-fire neurons and derive mesoscopic mean-field population equations, which reproduce the statistical and qualitative behavior of the homogeneous neuronal sub-populations. Similarly, Montbrió et al. (2015) derive a set of non-linear differential equations describing the dynamics of the rate and mean membrane potentials of a population of quadratic integrate-and-fire (QIF) neurons. The simulation platform PyRates (Gast et al., 2019) provides an implementation of this QIF mean-field model, and allows simulating them to obtain the temporal evolution of the population activity measures.

However, mean-field and related theories can go beyond such reduced dynamical equations: they can directly link model parameters to activity statistics, and they can even provide access to informative network properties that might not be accessible otherwise. The spectral bound (Rajan and Abbott, 2006) of the effective connectivity matrix in linear response theory (Lindner et al., 2005; Pernice et al., 2011; Trousdale et al., 2012) is an example of such a property. It is a measure for the stability of the linearized system and determines, for example, the occurrence of slow dynamics and long-range correlations (Dahmen et al., 2022). Another example is the sensitivity measure presented in Section 3.3.3, which directly links a network model's connectivity with the properties of its power spectrum. These measures are not accessible via simulations. They require analytical calculation.

Similarly, NNMT is not a simulator. NNMT is a collection of mean-field equation implementations that directly relate a model's parameters to the statistics of its dynamics or to other informative properties. It provides these implementations in a format that makes them applicable to as many network models as possible. This is not to say that NNMT does not involve numerical integration procedures; solving self-consistent equations, such as in the case of the firing rates calculations in Section 3.2.1 and Section 3.2.2, is a common task, and a collection of respective solvers is part of NNMT.

### 4.2. Use Cases

In Section 3, we present concrete examples of how to apply some of the tools available. Here, we revisit some of the examples to highlight the use cases NNMT lends itself to, as well as provide some ideas for how the toolbox could be utilized in future projects.

Analytical methods have the advantage of being fast, and typically they only require a limited amount of computational resources. The computational costs for calculating analytical estimates of dynamical network properties like firing rates, as opposed to the costs of running simulations of a network model, are independent of the number of neurons the network is composed of. This is especially relevant for parameter space explorations, for which many simulations have to be performed. To speed up prototyping, a modeler can first perform a parameter scan using analytical tools from NNMT to get an estimate of the right parameter regimes and subsequently run simulations on this restricted set of parameters to arrive at the final model parameters. An example of such a parameter scan is given in Section 3.2.1, where the firing rates of a network are studied as a function of the external input.

Additionally to speeding up parameter space explorations, analytical methods may guide parameter space explorations in another way: namely, by providing an analytical relation between network model parameters and network dynamics, which allows a targeted adjustment of specific parameters to achieve a desired network activity. The prime example implemented in NNMT is the sensitivity measure presented in Section 3.3.3, which provides an intuitive relation between the network connectivity and the peaks of the power spectrum corresponding to the dominant oscillation frequencies. As shown in the final part of Section 3.3.3, the sensitivity measure identifies the connections which need to be adjusted in order to modify the dominant oscillation mode in a desired manner. This illustrates a mean-field method that provides a modeler with additional information about the origin of a model's dynamics, such that a parameter space exploration can be restricted to the few identified crucial model parameters.

A modeler investigating which features of a network model are crucial for the emergence of certain activity characteristics observed in simulations might be interested in comparing models of differing complexity. The respective mappings can be derived in mean-field theory, and one variant included in NNMT, which is presented in Section 3.4, allows mapping a LIF network to a simpler rate network. This is useful to investigate whether spiking dynamics is crucial for the observed phenomenon.

On a general note, which kind of questions researchers pursue is limited by and therefore depends on the tools they have at hand (Dyson, 2012). The availability of sophisticated neural network simulators for example has lead to the development of conceptually new and more complex neural network models, precisely because their users could focus on actual research questions instead of implementations. We hope that collecting useful implementations of analytical tools for network model analysis will have a similar effect on the development of new tools and that it might lead to new, creative ways of applying them.

### 4.3. Limitations

As a collection of analytical methods, NNMT comes with inherent limitations that apply to any toolbox for analytical methods: it is restricted to network, neuron, and synapse models, as well as observables, for which a mean-field theory exists, and the tools are based on analytical assumptions, simplifications, and approximations, restricting their valid parameter regimes and their explanatory power, which we expand upon in the next paragraphs.

Analytical methods can provide good estimates of network model properties, but there are limitations that must be considered when interpreting results provided by NNMT: First of all, the employed numerical solvers introduce numerical inaccuracies, but they can be remedied by changing hyperparameters such as integration step sizes or iteration termination thresholds. More importantly, analytical methods almost always rely on approximations, which can only be justified if certain assumptions are fulfilled. Typical examples of such assumptions are fast or slow synapses, or a random connectivity. If such assumptions are not met, at least approximately, and the valid parameter regime of a tool is left, the corresponding method is not guaranteed to give reliable results. Hence, it is important to be aware of a tool's limitations, which we aim to document as thoroughly as possible.

An important assumption of mean-field theory is uncorrelated Poissonian inputs. As discussed in Section 3.2.1, asynchronous irregular activity is a robust feature of inhibition dominated networks, and mean-field theory is well-suited to describe the activity of such models. However, if a network model features highly correlated activity, or strong external input common to many neurons, approximating the input by uncorrelated noise no longer holds and mean-field estimates become unreliable.

In addition to the breakdown of such assumptions, some approaches, like linear response theory, rely on neglecting higher order terms. This restricts the tools' explanatory power, as they cannot predict higher order effects, such as the presence of higher harmonics in a network's power spectrum. Addressing these deficiencies necessitates using more elaborate analyses, and users should be aware of such limitations when interpreting the results.

Finally, a specific limitation of NNMT is that it currently only collects methods for LIF neurons. However, one of the aims of this paper is to encourage other scientists to contribute to the collection, and we outline how to do so in the following section.

### 4.4. How to Contribute and Outlook

A toolbox like NNMT always is an ongoing project, and there are various aspects that can be improved. In this section, we briefly discuss how available methods could be improved, what and how new tools could be added, as well as the benefits of implementing a new method with the help of NNMT.

First of all, NNMT in its current state is partly vectorized but the included methods are not parallelized, e.g., using multiprocessing or MPI for Python (mpi4py). Vectorization relies on NumPy (Harris et al., 2020) and SciPy (Virtanen et al., 2020), which are thread-parallel for specific backends, e.g., IntelMKL. With the tools available in the toolbox at the moment, run-time only becomes an issue in extensive parameter scans, for instance, when the transfer function needs to be calculated for a large range of frequencies. To further reduce the runtime, the code could be made fully vectorized. Alternatively, parallelization of many tools in NNMT is straightforward, as many of them include for loops over the different populations of a network model and for loops over the different analysis frequencies. A third option is just-in-time compilation, as provided by Numba (Lam et al., 2015).

Another aspect to consider is the range of network models a tool can be applied to. Thus far, the toolbox primarily supports arbitrary block structured networks. Future developments could extend the class of networks to even more general models.

Due to the research focus at our lab, NNMT presently mainly contains tools for LIF neurons in the fast synaptic regime and networks with random connectivity. Nonetheless, the structure of NNMT allows for adding methods for different neuron types, like for example binary (Ginzburg and Sompolinsky, 1994) or conductance-based neurons (Izhikevich, 2007; Richardson, 2007), as well as more elaborate network models. Another way to improve the toolbox is adding tools that complement the existing ones: As discussed in Section 4.3, many mean-field methods only give valid results for certain parameter ranges. Sometimes, there exist different approximations for the same quantity, valid in complementary parameter regimes. A concrete example is the currently implemented version of the transfer function for leaky integrate-and-fire neurons, based on Schuecker et al. (2015), which gives a good estimate for small synaptic time constants compared to the membrane time constant τ_s_/τ_m_ ≪ 1. A complementary estimate for τ_s_/τ_m_ ≫ 1 has been developed by Moreno-Bote and Parga (2006). Similarly, the current implementation of the firing rates of leaky integrate-and-fire neurons, based on the work of Fourcaud and Brunel (2002), is valid for τ_s_/τ_m_ ≪ 1. Recently, van Vreeswijk and Farkhooi (2019) have developed a method accurate for all combinations of synaptic and membrane time constants.

In the following, we explain how such implementations can be added and how using NNMT helps implementing new methods. Clearly, the implementations of NNMT help implementing methods that build on already existing ones. An example is the firing rate for LIF neurons with exponential synapses nnmt.lif.exp._firing_rates() which wraps the calculation of firing rates for LIF neurons with delta synapses nnmt.lif.delta._firing_rates(). Additionally, the toolbox may support the implementation of tools for other neuron models. As an illustration, let us consider adding the computation of the mean activity for a network of binary neurons (included in NNMT 1.1.0). We start with the equations for the mean input μ_*a*_, its variance $\sigma_{a}^{2}$, and the firing rates ***m*** (Helias et al., 2014, Equations 4, 6, and 7)


(19) $$\begin{array}{l} \mu_{a}\left(m\right)=\underset{b}{\sum }K_{ab}J_{ab}m_{b},\\ \sigma_{a}^{2}\left(m\right)=\underset{b}{\sum }K_{ab}J_{ab}^{2}m_{b}\left(1-m_{b}\right),\\ m_{a}\left(\mu_{a},\sigma_{a}\right)=\frac{1}{2}\text{erfc}\left(\frac{\Theta_{a}-\mu_{a}}{\sqrt{2}\sigma_{a}}\right), \end{array}$$

with indegree matrix *K*_*ab*_ from population *b* to population *a*, synaptic weight matrix *J*_*ab*_, and firing-threshold Θ_*a*_. The sum $\sum_{b}$ may include an external population providing input to the model. This set of self-consistent equations has the same structure as the self-consistent equations for the firing rates of a network of LIF neurons, Equation (8): the input statistics are given as functions of the rate, and the rate is given as a function of the input statistics. Therefore, it is possible to reuse the firing rate integration procedure for LIF neurons, providing immediate access to the two different methods presented in Section 3.2.1. Accordingly, it is sufficient to implement Equation (19) in a new submodule nnmt.binary and apply the solver provided by NNMT to extend the toolbox to binary neurons.

The above example demonstrates the benefits of collecting analytical tools for network model analysis in a common framework. The more methods and corresponding solvers the toolbox comprises, the easier implementing new methods becomes. Therefore, contributions to the toolbox are highly welcome; this can be done via the standard pull request workflow on GitHub (see the “Contributors guide” of the official documentation of NNMT^2^). We hope that in the future, many scientists will contribute to this collection of analytical methods for neuronal network model analysis, such that, at some point, we will have tools from all parts of mean-field theory of neuronal networks, made accessible in a usable format to all neuroscientists.

## Data Availability Statement

Publicly available datasets were used in this study, and the corresponding sources are cited in the main text. The toolbox's repository can be found at https://github.com/INM-6/nnmt, and the parameter files used in the presented examples can be found in the examples section of the online documentation https://nnmt.readthedocs.io/en/latest/.

## Author Contributions

HB and MH developed and implemented the code base and the initial version of the toolbox. ML, JS, and SE designed the current version of the toolbox. ML implemented the current version of the toolbox, vectorized and generalized tools, developed and implemented the test suite, wrote the documentation, and created the example shown in Section 3.2.2. AM improved the numerics of the firing rate integration (Methods) and created the example shown in Section 3.2.1. SE implemented integration tests, improved the functions related to the sensitivity_measure, and created the examples shown in Section 3.3. JS developed and implemented the tools used in Section 3.4 and created the respective example. ML, JS, SE, AM, and MH wrote this article. All authors approved the submitted version.

## Funding

This project has received funding from the European Union's Horizon 2020 Framework Programme for Research and Innovation under Specific Grant Agreement Nos. 720270 (HBP SGA1), 785907 (HBP SGA2), and 945539 (HBP SGA3), has been partially funded by the Deutsche Forschungsgemeinschaft (DFG, German Research Foundation) – 368482240/GRK2416, and has been partially funded by the Deutsche Forschungsgemeinschaft (DFG, German Research Foundation) – 491111487. This research was supported by the Joint Lab “Supercomputing and Modeling for the Human Brain”.

## Conflict of Interest

The authors declare that the research was conducted in the absence of any commercial or financial relationships that could be construed as a potential conflict of interest.

## Publisher's Note

All claims expressed in this article are solely those of the authors and do not necessarily represent those of their affiliated organizations, or those of the publisher, the editors and the reviewers. Any product that may be evaluated in this article, or claim that may be made by its manufacturer, is not guaranteed or endorsed by the publisher.

## Footnotes

^1^ https://github.com/INM-6/nnmt

^2^ https://nnmt.readthedocs.io/

^3^The Lambert *W*_*B*_ function is defined as $z=W_{B}\left(z\right){\text{e}}^{W_{B}\left(z\right)}$ for *z* ∈ ℂ and has infinitely many solutions, numbered by the branches *B*.

^4^ http://alleninstitute.github.io/dipde

## Appendix

### A.1. Siegert Implementation

Here, we describe how we solve the integral in Equation (4) numerically in a fully vectorized manner. The difficulty in Equation (4), $\phi \left(\mu ,\sigma \right)=1/\left[\tau_{\text{r}}+\tau_{\text{m}}\sqrt{\pi }I\left({\tilde{V}}_{0},{\tilde{V}}_{\text{th}}\right)\right]$ where ${\tilde{V}}_{0}={\tilde{V}}_{0}\left(\mu ,\sigma \right)$ and ${\tilde{V}}_{\text{th}}={\tilde{V}}_{\text{th}}\left(\mu ,\sigma \right)$ are determined by either Equation (5) or Equation (10), is posed by the integral

(A1)
$$\begin{array}{l} I\left({\tilde{V}}_{0},{\tilde{V}}_{\text{th}}\right)=\int_{{\tilde{V}}_{0}}^{{\tilde{V}}_{\text{th}}}e^{s^{2}}\left(1+\text{erf}\left(s\right)\right)\text{d}s. \end{array}$$

This integral is problematic due to the multiplication of *e*^*s*^2^^ and 1 + erf(*s*) in the integrand which leads to overflow and loss of significance.

To address this, we split the integral into different domains depending on the sign of the integration variable. Furthermore, we use the scaled complementary error function

(A2)
$$\begin{array}{l} \text{erf}\left(s\right)=1-e^{-s^{2}}\text{erfcx}\left(s\right) \end{array}$$

to extract the leading exponential contribution. Importantly, erfcx(*s*) decreases monotonically from erfcx(0) = 1 with power law asymptotics $\text{erfcx}\left(s\right)\sim 1/\left(\sqrt{\pi }s\right)$, hence it does not contain any exponential contribution. For positive *s*, the exponential contribution in the prefactor of erfcx(*s*) cancels the *e*^*s*^2^^ factor in the integrand. For negative *s*, the integrand simplifies even further to *e*^*s*^2^^(1 + erf(−*s*)) = erfcx(*s*) using erf(−*s*) = −erf(*s*). In addition to erfcx(*s*), we employ the Dawson function

(A3)
$$\begin{array}{l} D\left(s\right)=e^{-s^{2}}\int_{0}^{s}e^{r^{2}}\text{d}r \end{array}$$

to solve some of the integrals analytically. The Dawson function has a power law tail, *D*(*s*) ~ 1/(2*s*); hence, it also does not carry an exponential contribution. Both erfcx(*s*) and the Dawson function are fully vectorized in SciPy (Virtanen et al., 2020).

Any remaining integrals are solved using Gauss–Legendre quadrature (Press et al., 2007). By construction, Gauss–Legendre quadrature of order *k* solves integrals of polynomials up to degree *k* on the interval [−1, 1] exactly. Thus, it gives very good results if the integrand is well approximated by a polynomial of degree *k*. The quadrature rule itself is

(A4)
$$\begin{array}{ll} \int_{a}^{b}f\left(s\right)\text{d}s & \approx \frac{b-a}{2}\overset{k}{\underset{i=1}{\sum }}w_{i}f\left(\frac{b-a}{2}u_{i}+\frac{b+a}{2}\right), \end{array}$$

where the *u*_*i*_ are the roots of the Legendre polynomial of order *k* and the *w*_*i*_ are appropriate weights such that a polynomial of degree *k* is integrated exactly. We use a fixed order quadrature for which Equation (4) is straightforward to vectorize to multiple *a* and *b*. We determine the order of the quadrature iteratively by comparison with an adaptive quadrature rule; usually, a small order *k* = *O*(10) already yields very good results for an erfcx(*s*) integrand.

#### Inhibitory Regime

First, we consider the case where lower and upper bound of the integral are positive, $0<{\tilde{V}}_{0}<{\tilde{V}}_{\text{th}}$. This corresponds to strongly inhibitory mean input. Expressing the integrand in terms of erfcx(*s*) and using the Dawson function, we get

$$\begin{array}{l} I_{\text{inh}}\left({\tilde{V}}_{0},{\tilde{V}}_{\text{th}}\right)=2e^{{\tilde{V}}_{\text{th}}^{2}}D\left({\tilde{V}}_{\text{th}}\right)-2e^{{\tilde{V}}_{0}^{2}}D\left({\tilde{V}}_{0}\right)-\int_{{\tilde{V}}_{0}}^{{\tilde{V}}_{\text{th}}}\text{erfcx}\left(s\right)\text{d}s. \end{array}$$

The remaining integral is evaluated using Gauss–Legendre quadrature, Equation (4). We extract the leading contribution $e^{{\tilde{V}}_{\text{th}}^{2}}$ from the denominator in Equation (4) and arrive at

(A5)
$$\begin{array}{l} \phi \left(\mu ,\sigma \right)=\frac{e^{-{\tilde{V}}_{\text{th}}^{2}}}{\begin{array}{c} \tau_{\text{r}}e^{-{\tilde{V}}_{\text{th}}^{2}}+\tau_{\text{m}}\sqrt{\pi }\left(e^{-{\tilde{V}}_{\text{th}}^{2}}I_{\text{inh}}\left({\tilde{V}}_{0},{\tilde{V}}_{\text{th}}\right)\right) \end{array}}. \end{array}$$

Extracting $e^{{\tilde{V}}_{\text{th}}^{2}}$ from the denominator reduces the latter to $2\tau_{\text{m}}\sqrt{\pi }D\left({\tilde{V}}_{\text{th}}\right)$ and exponentially small correction terms (remember $0<{\tilde{V}}_{0}<{\tilde{V}}_{\text{th}}$ because *V*_0_ < *V*_th_), thereby preventing overflow.

#### Excitatory Regime

Second, we consider the case where lower and upper bound of the integral are negative, ${\tilde{V}}_{0}<{\tilde{V}}_{\text{th}}<0$. This corresponds to strongly excitatory mean input. In this regime, we change variables *s* → −*s* to make the domain of integration positive. Using erf(−*s*) = −erf(*s*) as well as erfcx(*s*), we get

$$\begin{array}{l} I_{\text{exc}}\left({\tilde{V}}_{0},{\tilde{V}}_{\text{th}}\right)=\int_{\left|{\tilde{V}}_{\text{th}}\right|}^{\left|{\tilde{V}}_{0}\right|}\text{erfcx}\left(s\right)\text{d}s. \end{array}$$

Thus, we evaluate Equation (4) as

(A6)
$$\begin{array}{l} \phi \left(\mu ,\sigma \right)=\frac{1}{\tau_{\text{r}}+\tau_{\text{m}}\sqrt{\pi }\int_{\left|{\tilde{V}}_{\text{th}}\right|}^{\left|{\tilde{V}}_{0}\right|}\text{erfcx}\left(s\right)\text{d}s}. \end{array}$$

In particular, there is no exponential contribution involved in this regime.

#### Intermediate Regime

Last, we consider the remaining case ${\tilde{V}}_{0}\leq 0\leq {\tilde{V}}_{\text{th}}$. We split the integral at zero and use the previous steps for the respective parts to get

$$\begin{array}{l} I_{\text{interm}}\left({\tilde{V}}_{0},{\tilde{V}}_{\text{th}}\right)=2e^{{\tilde{V}}_{\text{th}}^{2}}D\left({\tilde{V}}_{\text{th}}\right)+\int_{{\tilde{V}}_{\text{th}}}^{\left|{\tilde{V}}_{0}\right|}\text{erfcx}\left(s\right)\text{d}s. \end{array}$$

Note that the sign of the second integral depends on whether $|{\tilde{V}}_{0}|>{\tilde{V}}_{\text{th}}$ (+) or not (−). Again, we extract the leading contribution $e^{{\tilde{V}}_{\text{th}}^{2}}$ from the denominator in Equation (4) and arrive at

(A7)
$$\begin{array}{l} \phi \left(\mu ,\sigma \right)=\frac{e^{-{\tilde{V}}_{\text{th}}^{2}}}{\tau_{\text{r}}e^{-{\tilde{V}}_{\text{th}}^{2}}+\tau_{\text{m}}\sqrt{\pi }\left(e^{-{\tilde{V}}_{\text{th}}^{2}}I_{\text{interm}}\left({\tilde{V}}_{0},{\tilde{V}}_{\text{th}}\right)\right)}. \end{array}$$

As before, extracting $e^{{\tilde{V}}_{\text{th}}^{2}}$ from the denominator prevents overflow.

#### Deterministic Limit

The deterministic limit σ → 0 corresponds to $|{\tilde{V}}_{0}|,|{\tilde{V}}_{\text{th}}|\to \infty$ for both Equation (5) and Equation (10). In the inhibitory and the intermediate regime, we see immediately that ϕ(μ, σ → 0) → 0 due to the dominant contribution $e^{-{\tilde{V}}_{\text{th}}^{2}}$. In the excitatory regime, we use the asymptotics $\text{erfcx}\left(s\right)\sim 1/\left(\sqrt{\pi }s\right)$ to get

$$\begin{array}{ll} I\left({\tilde{V}}_{0},{\tilde{V}}_{\text{th}}\right) & \to \int_{\left|{\tilde{V}}_{\text{th}}\right|}^{\left|{\tilde{V}}_{0}\right|}\frac{1}{\sqrt{\pi }s}\text{d}s=\frac{1}{\sqrt{\pi }}\ln\frac{\left|{\tilde{V}}_{0}\right|}{\left|{\tilde{V}}_{\text{th}}\right|}. \end{array}$$

Inserting this into Equation (4) yields

(A8)
$$\begin{array}{ll} \phi \left(\mu ,\sigma \right) & \to \{\begin{array}{cc} \frac{1}{\tau_{\text{r}}+\tau_{\text{m}}\ln\frac{\mu -V_{0}}{\mu -V_{\text{th}}}} & \text{if }\mu >V_{\text{th}}\\ 0 & \text{otherwise} \end{array}, \end{array}$$

which is the firing rate of a leaky integrate-and-fire neuron driven by a constant input (Gerstner et al., 2014). Thus, this implementation also tolerates the deterministic limit of a very small noise intensity σ.

**Table A1:** Microcircuit Parameters.

**Symbol**	**Value (Potjans and Diesmann, 2014)**	**Value (Bos et al., 2016)**	**Description**
*K*_4E, 4I_	795	675	In-degree from 4I to 4E
*K*_4E, ext_	2100	1780	External in-degree to 4E
*D*(ω)	none	truncated Gaussian	Delay distribution
*d*_*e*_ ± δ*d*_*e*_	1.5 ± 0.75 ms	1.5 ± 1.5 ms	Mean and standard deviation of excitatory delay
*d*_*i*_ ± δ*d*_*i*_	0.75 ± 0.375 ms	0.75 ± 0.75 ms	Mean and standard deviation of inhibitory delay

*Parameter adaptions used here are introduced by Bos et al. (2016) compared to original microcircuit model. K_ij_ denotes the in-degrees from population j to population i. The delays in the simulated networks were drawn from a truncated Gaussian distribution with the given mean and standard deviation. The mean-field approximation of the microcircuit by Potjans and Diesmann (2014) assumes the delay to be fixed at the mean value, which is specified in the toolbox by setting the parameter delay_dist to none*.

### A.2. Transfer Function Notations

In Section 3.3.1 we introduce the analytical form of the transfer function implemented in the toolbox. Schuecker et al. (2015), derive a more general form of the transfer function, which includes a modulation of the variance of the input. Here we compare the notation used in Equation (11) to the notation used in Schuecker et al. (2015, Eq. 29).

Schuecker et al. (2015) define the modulations of input mean and variance as

(A9)
$$\begin{array}{l} \mu \left(t\right)=\mu +\epsilon \mu {\text{e}}^{\text{i}\omega t},\\ \sigma^{2}\left(t\right)=\sigma^{2}+H\sigma^{2}{\text{e}}^{\text{i}\omega t}, \end{array}$$

and introduce the transfer function in terms of its influence on the firing rate

$$\nu \left(t\right)/\nu_{0}=1+n\left(\omega \right){\text{e}}^{\text{i}\omega t},$$

where ν_0_ is the stationary firing rate. Here the transfer function *n*(ω) includes contributions of both the modulation of the mean *n*_*G*_(ω) ∝ ϵ and the modulation of the variance *n*_*H*_(ω) ∝ *H*. We write the modulation of the mean as

$$\mu \left(t\right)=\mu +\delta \mu {\text{e}}^{\text{i}\omega t},$$

implying that δμ corresponds to ϵμ in Equation (9). As we only consider the modulation of the mean, the firing rate can be rewritten as

$$\nu \left(t\right)=\nu +N\left(\omega \right)\delta \mu {\text{e}}^{\text{i}\omega t},$$

where we moved the stationary firing rate ν to the right hand side and included it in the definition of the transfer function *N*(ω). In the main text we emphasize that μ(*t*) and ν(*t*) are physical quantities by only considering the real part of complex contributions. Additionally, we swap the voltage boundaries in Equation (11), introducing a canceling sign change in both the numerator and the denominator. This reformulation was chosen to align the presented formula with the implementation in the toolbox.

## References

[B1] Abramowitz M. Stegun I. A. (1974). Handbook of Mathematical Functions: With Formulas, Graphs, and Mathematical Tables (New York: Dover Publications).

[B2] Ahmadian Y. Miller K. D. (2021). What is the dynamical regime of cerebral cortex? Neuron 109, 3373–3391. 10.1016/j.neuron.2021.07.031 34464597 PMC9129095

[B3] Amari S.-I. (1975). Homogeneous nets of neuron-like elements. Biol. Cybern. 17, 211–220. 10.1007/BF00339367 1125349

[B4] Amari S.-I. (1977). Dynamics of pattern formation in lateral-inhibition type neural fields. Biol. Cybern. 27, 77–87. 10.1007/bf00337259 911931

[B5] Amit D. J. Brunel N. (1997a). Dynamics of a recurrent network of spiking neurons before and following learning. Netw. Comp. Neural Sys. 8, 373–404. 10.1088/0954-898x_8_4_00326463272

[B6] Amit D. J. Brunel N. (1997b). Model of global spontaneous activity and local structured activity during delay periods in the cerebral cortex. Cereb. Cortex 7, 237–252. 10.1093/cercor/7.3.237 9143444

[B7] Amit D. J. Tsodyks M. V. (1991). Quantitative study of attractor neural network retrieving at low spike rates I: substrate–spikes, rates and neuronal gain. Network 2, 259. 10.1088/0954-898X_2_3_003

[B8] Bos H. Diesmann M. Helias M. (2016). Identifying anatomical origins of coexisting oscillations in the cortical microcircuit. PLOS Comput. Biol. 12, e1005132. 10.1371/journal.pcbi.1005132 27736873 PMC5063581

[B9] Braitenberg V. Schüz A. (1998). Cortex: Statistics and Geometry of Neuronal Connectivity, 2nd Edn. Berlin: Springer-Verlag.

[B10] Bressloff P. C. (2012). Spatiotemporal dynamics of continuum neural fields. J. Phys. A 45, 033001. 10.1088/1751-8113/45/3/03300120457860

[B11] Bressloff P. C. Cowan J. D. Golubitsky M. Thomas P. J. Wiener M. C. (2001). Geometric visual hallucinations, euclidean symmetry and the functional architecture of striate cortex. Phil. Trans. R. Soc. B 356, 299–330. 10.1098/rstb.2000.0769 11316482 PMC1088430

[B12] Brunel N. (2000). Dynamics of sparsely connected networks of excitatory and inhibitory spiking neurons. J. Comput. Neurosci. 8, 183–208. 10.1023/a:1008925309027 10809012

[B13] Brunel N. Chance F. S. Fourcaud N. Abbott L. F. (2001). Effects of synaptic noise and filtering on the frequency response of spiking neurons. Phys. Rev. Lett. 86, 2186–2189. 10.1103/physrevlett.86.2186 11289886

[B14] Brunel N. Hakim V. (1999). Fast global oscillations in networks of integrate-and-fire neurons with low firing rates. Neural Comput. 11, 1621–1671. 10.1162/089976699300016179 10490941

[B15] Brunel N. Latham P. (2003). Firing rate of the noisy quadratic integrate-and-fire neuron. Neural Comput. 15, 2281–2306. 10.1162/089976603322362365 14511522

[B16] Buice M. A. Chow C. C. (2013). Beyond mean field theory: statistical field theory for neural networks. J. Stat. Mech. 2013, P03003. 10.1088/1742-5468/2013/03/P03003 25243014 PMC4169078

[B17] Coombes S. (2005). Waves, bumps, and patterns in neural field theories. Biol. Cybern. 93, 91–108. 10.1007/s00422-005-0574-y 16059785

[B18] Coombes S. bei Graben P. Potthast R. Wright J. (2014). Neural Fields. Theory and Applications. Berlin; Heidelberg: Springer-Verlag.

[B19] Corless R. M. Gonnet G. H. Hare D. E. G. Jeffrey D. J. Knuth D. E. (1996). On the lambert w function. Adv. Comput. Math. 5, 329–359. 10.1007/BF02124750

[B20] Dahmen D. Layer M. Deutz L. Dąbrowska P. A. Voges N. von Papen M.. (2022). Global organization of neuronal activity only requires unstructured local connectivity. eLife 11, e68422. 10.7554/eLife.68422.sa0 35049496 PMC8776256

[B21] Dasbach S. Tetzlaff T. Diesmann M. Senk J. (2021). Dynamical characteristics of recurrent neuronal networks are robust against low synaptic weight resolution. Front. Neurosci. 15, 757790. 10.3389/fnins.2021.757790 35002599 PMC8740282

[B22] DeFelipe J. Alonso-Nanclares L. Arellano J. (2002). Microstructure of the neocortex: comparative aspects. J. Neurocytol. 31, 299–316. 10.1023/A:1024130211265 12815249

[B23] Doedel E. J. Oldeman B. (1998). Auto-07p: Continuation and Bifurcation Software. Montreal, QC: Concordia University Canada

[B24] Dyson F. J. (2012). Is science mostly driven by ideas or by tools? Science 338, 1426–1427. 10.1126/science.1232773 23239721

[B25] Ermentrout B. (2002). Simulating, Analyzing, and Animating Dynamical Systems: A Guide to Xppaut for Researchers and Students (Software, Environments, Tools). Philadelphia, PA: Society for Industrial and Applied Mathematics.

[B26] Ermentrout G. B. Cowan J. D. (1979). A mathematical theory of visual hallucination patterns. Biol. Cybern. 34, 137–150. 10.1007/BF00336965 486593

[B27] Fourcaud N. Brunel N. (2002). Dynamics of the firing probability of noisy integrate-and-fire neurons. Neural Comput. 14, 2057–2110. 10.1162/089976602320264015 12184844

[B28] Fourcaud-Trocmé N. Hansel D. van Vreeswijk C. Brunel N. (2003). How spike generation mechanisms determine the neuronal response to fluctuating inputs. J. Neurosci. 23, 11628–11640. 10.1523/JNEUROSCI.23-37-11628.2003 14684865 PMC6740955

[B29] Gast R. Rose D. Salomon C. Möller H. E. Weiskopf N. Knösche T. R. (2019). Pyrates - a python framework for rate-based neural simulations. PLoS ONE 14, e0225900. 10.1371/journal.pone.0225900 31841550 PMC6913930

[B30] Gerstner W. Kistler W. M. Naud R. Paninski L. (2014). Neuronal Dynamics. From Single Neurons to Networks and Models of Cognition. Cambridge: Cambridge University Press.

[B31] Gewaltig M.-O. Diesmann M. (2007). NEST (nEural simulation tool). Scholarpedia 2, 1430. 10.4249/scholarpedia.1430

[B32] Giese M. A. (2012). Dynamic Neural Field Theory for Motion Perception, Vol. 469. Berlin; Heidelberg: Springer Science & Business Media)

[B33] Ginzburg I. Sompolinsky H. (1994). Theory of correlations in stochastic neural networks. Phys. Rev. E 50, 3171–3191. 10.1103/PhysRevE.50.3171 9962363

[B34] Goldenfeld N. (1992). Lectures on Phase Transitions and the Renormalization Group. Reading, MA: Perseus books.

[B35] Golosio B. Tiddia G. Luca C. D. Pastorelli E. Simula F. Paolucci P. S. (2021). Fast simulations of highly-connected spiking cortical models using GPUs. Front. Comput. Neurosci. 15, 627620. 10.3389/fncom.2021.627620 33679358 PMC7925400

[B36] Grabska-Barwinska A. Latham P. (2014). How well do mean field theories of spiking quadratic-integrate-and-fire networks work in realistic parameter regimes? J. Comput. Neurosci. 36, 469–481. 10.1007/s10827-013-0481-5 24091644

[B37] Grytskyy D. Tetzlaff T. Diesmann M. Helias M. (2013). A unified view on weakly correlated recurrent networks. Front. Comput. Neurosci. 7, 131. 10.3389/fncom.2013.00131 24151463 PMC3799216

[B38] Hagen E. Dahmen D. Stavrinou M. L. Lindén H. Tetzlaff T. van Albada S. J.. (2016). Hybrid scheme for modeling local field potentials from point-neuron networks. Cereb. Cortex 26, 4461–4496. 10.1093/cercor/bhw237 27797828 PMC6193674

[B39] Harris C. R. Millman K. J. van der Walt S. J. Gommers R. Virtanen P. Cournapeau D.. (2020). Array programming with NumPy. Nature 585, 357–362. 10.1038/s41586-020-2649-2 32939066 PMC7759461

[B40] Heitmann S. Aburn M. J. Breakspear M. (2018). The brain dynamics toolbox for matlab. Neurocomputing 315, 82–88. 10.1016/j.neucom.2018.06.026

[B41] Helias M. Tetzlaff T. Diesmann M. (2014). The correlation structure of local cortical networks intrinsically results from recurrent dynamics. PLoS Comput. Biol. 10, e1003428. 10.1371/journal.pcbi.1003428 24453955 PMC3894226

[B42] Hertz J. (2010). Cross-correlations in high-conductance states of a model cortical network. Neural Comput. 22, 427–447. 10.1162/neco.2009.06-08-806 19842988

[B43] Hines M. L. Carnevale N. T. (2001). NEURON: a tool for neuroscientists. Neuroscientist 7, 123–135. 10.1177/107385840100700207 11496923

[B44] Izhikevich E. M. (2007). Dynamical Systems in Neuroscience: The Geometry of Excitability and Bursting. Cambridge, MA: MIT Press.

[B45] Jirsa V. K. Haken H. (1996). Field theory of electromagnetic brain activity. Phys. Rev. Lett. 77, 960. 10.1103/PhysRevLett.77.960 10062950

[B46] Jirsa V. K. Haken H. (1997). A derivation of a macroscopic field theory of the brain from the quasi-microscopic neural dynamics. Phys. D 99, 503–526. 10.1016/S0167-2789(96)00166-2

[B47] Knight J. C. Nowotny T. (2018). GPUs outperform current HPC and neuromorphic solutions in terms of speed and energy when simulating a highly-connected cortical model. Front. Neurosci. 12, 941. 10.3389/fnins.2018.00941 30618570 PMC6299048

[B48] Laing C. R. Troy W. C. (2003). Two-bump solutions of amari-type models of neuronal pattern formation. Phys. D 178, 190–218. 10.1016/S0167-2789(03)00013-734417880

[B49] Laing C. R. Troy W. C. Gutkin B. Ermentrout B. G. (2002). Multiple bumps in a neuronal model of working memory. SIAM J. Appl. Math. 63, 62–97. 10.1137/s0036139901389495

[B50] Lam S. K. Pitrou A. Seibert S. (2015). Numba: a llvm-based python jit compiler, in Proceedings of the Second Workshop on the LLVM Compiler Infrastructure in HPC, Austin, TX, 1–6

[B51] Layer M. Senk J. Essink S. van Meegen A. Bos H. Helias M. (2021). NNMT (1.0.0). Zenodo. 10.5281/zenodo.5779548 PMC9196133 35712677

[B52] Lindner B. (2004). Interspike interval statistics of neurons driven by colored noise. Phys. Rev. E 69, 0229011–0229014. 10.1103/PhysRevE.69.022901 14995506

[B53] Lindner B. Doiron B. Longtin A. (2005). Theory of oscillatory firing induced by spatially correlated noise and delayed inhibitory feedback. Phys. Rev. E 72, 061919. 10.1103/physreve.72.061919 16485986

[B54] Lindner B. Longtin A. (2005). Effect of an exponentially decaying threshold on the firing statistis of a stochastic integate-and-fire neuron. J. Theor. Biol. 232, 505–521. 10.1016/j.jtbi.2004.08.030 15588632

[B55] Lindner B. Schimansky-Geier L. (2001). Transmission of noise coded versus additive signals through a neuronal ensemble. Phys. Rev. Lett. 86, 2934–2937. 10.1103/physrevlett.86.2934 11290076

[B56] Mattia M. Biggio M. Galluzzi A. Storace M. (2019). Dimensional reduction in networks of non-markovian spiking neurons: Equivalence of synaptic filtering and heterogeneous propagation delays. PLoS Comput. Biol. 15, e1007404. 10.1371/journal.pcbi.1007404 31593569 PMC6799936

[B57] Montbrió E. Pazó D. Roxin A. (2015). Macroscopic description for networks of spiking neurons. Phys Rev X 5, 021028. 10.1103/PhysRevX.5.021028

[B58] Moreno-Bote R. Parga N. (2006). Auto- and crosscorrelograms for the spike response of leaky integrate-and-fire neurons with slow synapses. Phys. Rev. Lett. 96, 028101. 10.1103/PhysRevLett.96.028101 16486646

[B59] Nunez P. L. (1974). The brain wave equation: a model for the eeg. Math. Biosci. 21, 279–297. 10.1016/0025-5564(74)90020-0

[B60] Olver F. W. J. Olde Daalhuis A. B. Lozier D. W. Schneider B. I. Boisvert R. F. Clark C. W.. (2021). NIST Digital Library of Mathematical Functions. Available online at: http://dlmf.nist.gov/

[B61] Ostojic S. (2014). Two types of asynchronous activity in networks of excitatory and inhibitory spiking neurons. Nat. Neurosci. 17, 594–600. 10.1038/nn.3658 24561997

[B62] Ostojic S. Brunel N. (2011). From spiking neuron models to linear-nonlinear models. PLoS Comput. Biol. 7, e1001056. 10.1371/journal.pcbi.1001056 21283777 PMC3024256

[B63] Pernice V. Staude B. Cardanobile S. Rotter S. (2011). How structure determines correlations in neuronal networks. PLoS Comput. Biol. 7, e1002059. 10.1371/journal.pcbi.1002059 21625580 PMC3098224

[B64] Potjans T. C. Diesmann M. (2014). The cell-type specific cortical microcircuit: relating structure and activity in a full-scale spiking network model. Cereb. Cortex 24, 785–806. 10.1093/cercor/bhs358 23203991 PMC3920768

[B65] Press W. H. Teukolsky S. A. Vetterling W. T. Flannery B. P. (2007). Numerical Recipes: The Art of Scientific Computing, 3rd edn. Cambridge University Press.

[B66] Rajan K. Abbott L. F. (2006). Eigenvalue spectra of random matrices for neural networks. Phys. Rev. Lett. 97, 188104. 10.1103/PhysRevLett.97.188104 17155583

[B67] Renart A. De La Rocha J. Bartho P. Hollender L. Parga N. Reyes A.. (2010). The asynchronous state in cortical circuits. Science 327, 587–590. 10.1126/science.1179850 20110507 PMC2861483

[B68] Richardson M. J. E. (2007). Firing-rate response of linear and nonlinear integrate-and-fire neurons to modulated current-based and conductance-based synaptic drive. Phys. Rev. E 76, 1–15. 10.1103/PhysRevE.76.021919 17930077

[B69] Richardson M. J. E. (2008). Spike-train spectra and network response functions for non-linear integrate-and-fire neurons. Biol. Cybern. 99, 381–392. 10.1007/s00422-008-0244-y 19011926

[B70] Riquelme J. L. Gjorgjieva J. (2021). Towards readable code in neuroscience. Nat. Rev. Neurosci. 22, 257–258. 10.1038/s41583-021-00450-y 33707744

[B71] Rosenbaum R. Doiron B. (2014). Balanced networks of spiking neurons with spatially dependent recurrent connections. Phys. Rev. X 4, 021039. 10.1103/PhysRevX.4.021039

[B72] Rosenbaum R. Smith M. A. Kohn A. Rubin J. E. Doiron B. (2017). The spatial structure of correlated neuronal variability. Nat. Neurosci. 20, 107–114. 10.1038/nn.4433 27798630 PMC5191923

[B73] Sanz Leon P. Knock S. Woodman M. Domide L. Mersmann J. McIntosh A.. (2013). The virtual brain: a simulator of primate brain network dynamics. Front. Neuroinform. 7, 10. 10.3389/fninf.2013.00010 23781198 PMC3678125

[B74] Sanzeni A. Histed M. H. Brunel N. (2020). Response nonlinearities in networks of spiking neurons. PLOS Comput. Biol. 16, e1008165. 10.1371/journal.pcbi.1008165 32941457 PMC7524009

[B75] Schmidt M. Bakker R. Hilgetag C. C. Diesmann M. van Albada S. J. (2018). Multi-scale account of the network structure of macaque visual cortex. Brain Struct. Func. 223, 1409–1435. 10.1007/s00429-017-1554-4 29143946 PMC5869897

[B76] Schöner G. (2008). Dynamical systems approaches to cognition, in Cambridge Handbook of Computational Cognitive Modeling. Cambridge: Cambridge University Press, 101–126.

[B77] Schuecker J. Diesmann M. Helias M. (2014). Reduction of colored noise in excitable systems to white noise and dynamic boundary conditions. arXiv[Preprint].arXiv:1410.8799. 10.48550/arXiv.1410.8799

[B78] Schuecker J. Diesmann M. Helias M. (2015). Modulated escape from a metastable state driven by colored noise. Phys. Rev. E 92, 052119. 10.1103/PhysRevE.92.052119 26651659

[B79] Schuecker J. Goedeke S. Helias M. (2018). Optimal sequence memory in driven random networks. Phys. Rev. X 8, 041029. 10.1103/PhysRevX.8.041029

[B80] Schwalger T. Deger M. Gerstner W. (2017). Towards a theory of cortical columns: From spiking neurons to interacting neural populations of finite size. PLoS Comput. Biol. 13, e1005507. 10.1371/journal.pcbi.1005507 28422957 PMC5415267

[B81] Schwalger T. Droste F. Lindner B. (2015). Statistical structure of neural spiking under non-poissonian or other non-white stimulation. J. Comput. Neurosci. 39, 29. 10.1007/s10827-015-0560-x 25936628

[B82] Sejnowski T. (1976). On the stochastic dynamics of neuronal interaction. Biol. Cybern. 22, 203–211. 10.1007/BF00365086 953078

[B83] Senk J. Korvasová K. Schuecker J. Hagen E. Tetzlaff T. Diesmann M.. (2020). Conditions for wave trains in spiking neural networks. Phys. Rev. Res. 2, 023174. 10.1103/physrevresearch.2.023174

[B84] Senk J. Kriener B. Djurfeldt M. Voges N. Jiang H.-J. Schüttler L. (in press). Connectivity concepts in neuronal network modeling. PLOS Comput. Biol. 10.1371/journal.pcbi.1010086 PMC9455883 36074778

[B85] Sherfey J. S. Soplata A. E. Ardid S. Roberts E. A. Stanley D. A. Pittman-Polletta B. R.. (2018). Dynasim: a matlab toolbox for neural modeling and simulation. Front. Neuroinform. 12, 10. 10.3389/fninf.2018.00010 29599715 PMC5862864

[B86] Siegert A. J. (1951). On the first passage time probability problem. Phys. Rev. 81, 617–623. 10.1103/PhysRev.81.617

[B87] Sompolinsky H. Crisanti A. Sommers H. J. (1988). Chaos in random neural networks. Phys. Rev. Lett. 61, 259–262. 10.1103/PhysRevLett.61.259 10039285

[B88] Stiller J. Radons G. (1998). Dynamics of nonlinear oscillators with random interactions. Phys. Rev. E 58, 1789. 10.1103/PhysRevE.58.1789

[B89] Stimberg M. Brette R. Goodman D. F. (2019). Brian 2, an intuitive and efficient neural simulator. eLife 8, e47314. 10.7554/elife.47314 31429824 PMC6786860

[B90] Tetzlaff T. Helias M. Einevoll G. T. Diesmann M. (2012). Decorrelation of neural-network activity by inhibitory feedback. PLOS Comput. Biol. 8, e1002596. 10.1371/journal.pcbi.1002596 23133368 PMC3487539

[B91] Toyoizumi T. Abbott L. F. (2011). Beyond the edge of chaos: Amplification and temporal integration by recurrent networks in the chaotic regime. Phys. Rev. E 84, 051908. 10.1103/PhysRevE.84.051908 22181445 PMC5558624

[B92] Trousdale J. Hu Y. Shea-Brown E. Josic K. (2012). Impact of network structure and cellular response on spike time correlations. PLoS Comput. Biol. 8, e1002408. 10.1371/journal.pcbi.1002408 22457608 PMC3310711

[B93] Tuckwell H. C. (1988). Introduction to Theoretical Neurobiology, Vol. 2 Cambridge: Cambridge University Press.

[B94] van Albada S. J. Rowley A. G. Senk J. Hopkins M. Schmidt M. Stokes A. B.. (2018). Performance comparison of the digital neuromorphic hardware SpiNNaker and the neural network simulation software NEST for a full-scale cortical microcircuit model. Front. Neurosci. 12, 291. 10.3389/fnins.2018.00291 29875620 PMC5974216

[B95] van Meegen A. Lindner B. (2018). Self-consistent correlations of randomly coupled rotators in the asynchronous state. Phys. Rev. Lett. 121, 258302. 10.1103/PhysRevLett.121.258302 30608814

[B96] van Vreeswijk C. Farkhooi F. (2019). Fredholm theory for the mean first-passage time of integrate-and-fire oscillators with colored noise input. Phys. Rev. E 100, 060402. 10.1103/PhysRevE.100.060402 31962454

[B97] van Vreeswijk C. Sompolinsky H. (1996). Chaos in neuronal networks with balanced excitatory and inhibitory activity. Science 274, 1724–1726. 10.1126/science.274.5293.1724 8939866

[B98] van Vreeswijk C. Sompolinsky H. (1998). Chaotic balanced state in a model of cortical circuits. Neural Comput. 10, 1321–1371. 10.1162/089976698300017214 9698348

[B99] Virtanen P. Gommers R. Oliphant T. E. Haberland M. Reddy T. Cournapeau D.. (2020). SciPy 1.0: fundamental algorithms for scientific computing in python. Nat. Methods 17, 261–272. 10.1038/s41592-019-0686-2 32015543 PMC7056644

[B100] Wagatsuma N. Potjans T. C. Diesmann M. Fukai T. (2011). Layer-dependent attentional processing by top-down signals in a visual cortical microcircuit model. Front. Comput. Neurosci. 5, 31. 10.3389/fncom.2011.00031 21779240 PMC3134838

[B101] Wilson H. R. Cowan J. D. (1972). Excitatory and inhibitory interactions in localized populations of model neurons. Biophys. J. 12, 1 – 24. 10.1016/S0006-3495(72)86068-5 4332108 PMC1484078

[B102] Wilson H. R. Cowan J. D. (1973). A mathematical theory of the functional dynamics of cortical and thalamic nervous tissue. Kybernetik 13, 55–80. 10.1007/BF00288786 4767470

